# The Multifaceted Antibacterial Mechanisms of the Pioneering Peptide Antibiotics Tyrocidine and Gramicidin S

**DOI:** 10.1128/mBio.00802-18

**Published:** 2018-10-09

**Authors:** Michaela Wenzel, Marina Rautenbach, J. Arnold Vosloo, Tjalling Siersma, Christopher H. M. Aisenbrey, Ekaterina Zaitseva, Wikus E. Laubscher, Wilma van Rensburg, Jan C. Behrends, Burkhard Bechinger, Leendert W. Hamoen

**Affiliations:** a Bacterial Cell Biology, Swammerdam Institute for Life Sciences, University of Amsterdam, Amsterdam, The Netherlands; b Department of Medical Microbiology and Infection Control, Amsterdam University Medical Centers, Location VUMC, Amsterdam, The Netherlands; c BIOPEP Peptide Group, Department of Biochemistry, Faculty of Science, Stellenbosch University, Stellenbosch, South Africa; d Chemistry Institute UMR7177, University of Strasbourg/CNRS, Strasbourg, France; e Department of Physiology, Laboratory for Membrane Physiology and Technology, University of Freiburg, Freiburg, Germany; Utrecht University; Nanyang Technological University

**Keywords:** antibiotics, antimicrobial peptides, bacterial cell biology, bacterial cytological profiling, cell membranes, mode of action

## Abstract

Cyclic β-sheet decapeptides from the tyrocidine group and the homologous gramicidin S were the first commercially used antibiotics, yet it remains unclear exactly how they kill bacteria. We investigated their mode of action using a bacterial cytological profiling approach. Tyrocidines form defined ion-conducting pores, induce lipid phase separation, and strongly reduce membrane fluidity, resulting in delocalization of a broad range of peripheral and integral membrane proteins. Interestingly, they also cause DNA damage and interfere with DNA-binding proteins. Despite sharing 50% sequence identity with tyrocidines, gramicidin S causes only mild lipid demixing with minor effects on membrane fluidity and permeability. Gramicidin S delocalizes peripheral membrane proteins involved in cell division and cell envelope synthesis but does not affect integral membrane proteins or DNA. Our results shed a new light on the multifaceted antibacterial mechanisms of these antibiotics and explain why resistance to them is virtually nonexistent.

## Abstract (precis)

Cyclic β-sheet decapeptides, such as tyrocidines and gramicidin S, were among the first antibiotics in clinical application. Although they have been used for such a long time, there is virtually no resistance to them, which has led to a renewed interest in this peptide class. Both tyrocidines and gramicidin S are thought to disrupt the bacterial membrane. However, this knowledge is mainly derived from *in vitro* studies, and there is surprisingly little knowledge about how these long-established antibiotics kill bacteria. Our results shed new light on the antibacterial mechanism of β-sheet peptide antibiotics and explain why they are still so effective and why there is so little resistance to them.

## INTRODUCTION

**FIG 1:**
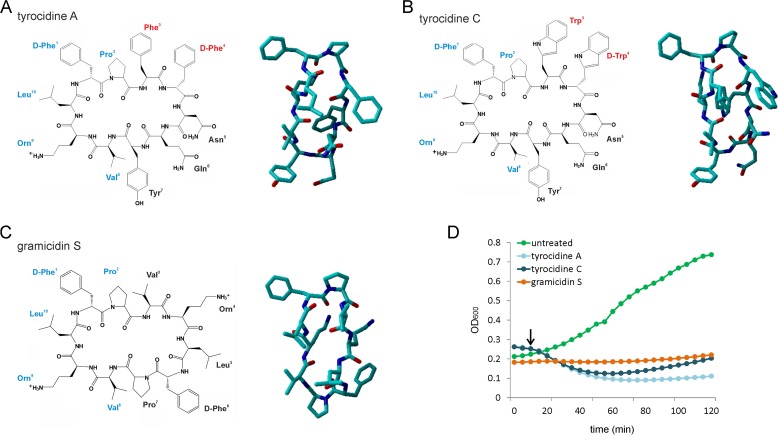
Peptides used in this study and their effects on the growth of B. subtilis. (A to C) Tyrocidine A (A), tyrocidine C (B), and gramicidin S (C) (left panels, skeletal structure models; right panels, stick models). Tyrocidines consist of a conserved cyclic decapeptide structure of the consensus sequence cyclo(d-Phe^1^-Pro^2^-X^3^-d-X^4^-Asn^5^-Gln^6^-Tyr^7^-Val^8^-Orn^9^-Leu^10^). Analogues of the tyrocidines containing either Phe^3^-d-Phe^4^, Trp^3^-d-Phe^4^, or Trp^3^-d-Trp^4^ at the variable dipeptide unit X^3^-d-X^4^ (red) are referred to as tyrocidine A, B, or C, respectively. Gramicidin S [cyclo(Val^8^-Orn^9^-Leu^10^-d-Phe^1^-Pro^2^)_2_] is composed of two repeat moieties of the conserved pentapeptide sequence of the tyrocidines (blue). Three-dimensional structures of the peptides are derived from the work of Munyuki et al. (93) (tyrocidines) and Stern et al. (99) (gramicidin S). (D) Growth of B. subtilis exposed to 1× MIC of the peptides (5.4 µg/ml tyrocidine A, 2.7 µg/ml tyrocidine C, and 1 µg/ml gramicidin S). Cells were grown until early exponential phase prior to addition of compounds. The arrow indicates the time point chosen for further mode-of-action analysis (10 min).

Cyclic β-sheet peptides produced by soil bacilli were among the very first antibiotic substances in clinical application and are still used today. One of the most important pioneering antibiotics was tyrothricin, a mixture of polypeptide antibiotics produced by Brevibacillus parabrevis (1). Although discovered 10 years after penicillin, tyrothricin was actually the first antibiotic preparation that was commercially produced for clinical use, 2 years before penicillin became commercially available. In fact, the success of tyrothricin inspired the reinvestigation of penicillin as a potential antibiotic drug, after penicillin had been deemed unstable, difficult to produce, and of no medical interest (2). Two years after the commercialization of tyrothricin, gramicidin S was discovered in the Soviet Union, where it was extensively used to treat gunshot wounds inflicted on the battlefields of World War II (3). Gramicidin S belongs to the same antibiotic class as tyrothricin but is produced by Aneurinibacillus migulanus (4). Both compounds display strong hemolytic potential and are limited to topical applications but are still used today, e.g., for superficial skin and throat infections (5–10). The imminent public health threat posed by multidrug-resistant microbial pathogens has rejuvenated investigations of these topical antibiotics for new medical applications. One of the major components of tyrothricin is the tyrocidines, cyclic β-sheet decapeptides (Fig. 1A and B) (4) with potent activity against several important pathogens, including the Gram-positive bacterium Listeria monocytogenes (11, 12), the pathogenic fungus Aspergillus fumigatus (13) and yeast Candida albicans (14), and the human malaria parasite Plasmodium falciparum (15). Thus, they have promising potential for broader applications than the current usage of tyrothricin. Gramicidin S, which shares 50% sequence homology with the tyrocidines (Fig. 1C), is highly active against Gram-positive staphylococci and enterococci, as well as Gram-negative Escherichia coli and Pseudomonas aeruginosa (16). This remarkable potency, and the fact that there is virtually no resistance to these old peptide antibiotics (17), has sparked a renewed interest in extending their clinical applications (9, 18), and considerable efforts have been undertaken to develop analogues with lower hemolytic potential (12, 19–25).

Despite this renewed attention, there is very little information on how exactly tyrocidines kill bacteria (15, 26). Based on an early *in vitro* study using artificial liposomes, it is assumed that tyrocidines permeabilize membranes, induce lipid phase separation, and increase fluidity of the hydrocarbon part of the lipid bilayer (26). How these effects are achieved and whether these observations are relevant for the *in vivo* situation are unknown. Studies with L. monocytogenes and the filamentous plant fungi Fusarium solani and Botrytis cinerea confirmed membrane permeabilization by tyrocidines (11, 27). However, there is evidence that these peptides also target β-glucans in the fungal cell wall (27), suggesting that there is more to their antimicrobial activity than only membrane disruption. Importantly, there is almost no further information available on how these peptides act on bacterial cells, although they have been clinically used against bacterial infections since 1940 (2). Early studies showed a reduced oxygen consumption in tyrocidine-treated Staphylococcus aureus (28), and inhibition of certain NADH-utilizing enzymes of the tyrothricin producer strain (29), but this has not been followed up.

In contrast to the tyrocidines, the interaction of gramicidin S with model membrane systems has been well investigated, and the consensus is that the drug partitions into the membrane at the interface between phospholipid head groups and fatty acid chains, thereby disturbing lipid packing, leading to membrane collapse at high concentrations (30–32). However, there is an ongoing dispute on whether it forms discrete pores or destroys the membrane in a detergent-like manner and whether membrane permeabilization occurs at all at concentrations relevant for bacterial killing (31, 33–37). Unfortunately, since the majority of early studies on gramicidin S were performed in the middle of the 20th century in the Soviet Union, most reports are available only in Russian and not easily accessible to the international scientific community. One of these early studies described inhibition of proteins in the membrane of its producer strain (38), and recently, it was shown that gramicidin S interferes with the membrane binding of the cell wall synthesis enzyme MurG and the electron transport chain protein cytochrome *c* (39). It has also been reported that both gramicidin S and the tyrocidines bind to DNA *in vitro*, suggesting an alternative or additional killing mechanism (40–43).

The structural homology of gramicidin S and tyrocidines suggests that these peptide antibiotics kill bacteria in the same way. To investigate this, we performed an *in vivo* mode-of-action study employing a recently established bacterial cytological profiling method that makes use of a broad array of fluorescently labeled proteins (44). This study revealed that tyrocidines and gramicidin S kill bacteria by surprisingly different mechanisms.

## RESULTS

### Cell wall integrity.

To assess whether the variable aromatic dipeptide unit of the tyrocidine peptides makes a difference for their *in vivo* mechanism, we used both tyrocidine A (Phe^3^-d-Phe^4^) and tyrocidine C (Trp^3^-d-Trp^4^) (Fig. 1A and B). The tyrocidines have been proposed to bind to fungal cell wall components (27), and gramicidin S was shown to affect cell wall integrity in Bacillus subtilis (37). To study how effectively these antibiotics weaken the bacterial cell wall and cause lysis, we followed the optical density (OD) of B. subtilis cells treated with MICs of the compounds (Fig. 1D). At this concentration, a fraction of the tyrocidine-treated cells lysed after 15 to 20 min of treatment but the culture recovered after approximately 60 min. Gramicidin S treatment did not lead to cell lysis but completely halted further growth. However, at 2× MIC, all three peptides caused lysis and no regrowth could be observed (see Fig. S1 in the supplemental material). Such cell lysis suggests that these antibiotics somehow affect the integrity of the cell wall. Since we wanted to analyze the immediate growth-inhibiting effects of the peptides, instead of pleiotropic lysis effects, we performed further experiments after 10 min of treatment with 1× MIC (unless otherwise noted), when no strong reduction of the optical density was observed (Fig. 1D).

10.1128/mBio.00802-18.3 FIG S1 Growth of B. subtilis 168 treated with 2× MIC of tyrocidine A (10.8 µg/ml), tyrocidine C (5.4 µg/ml), and gramicidin S (2 µg/ml). Cells were grown until early exponential phase prior to addition of compounds. Download FIG S1, TIF file, 0.2 MB. Copyright © 2018 Wenzel et al. 2018 Wenzel et al. This content is distributed under the terms of the Creative Commons Attribution 4.0 International license.

**FIG 2:**
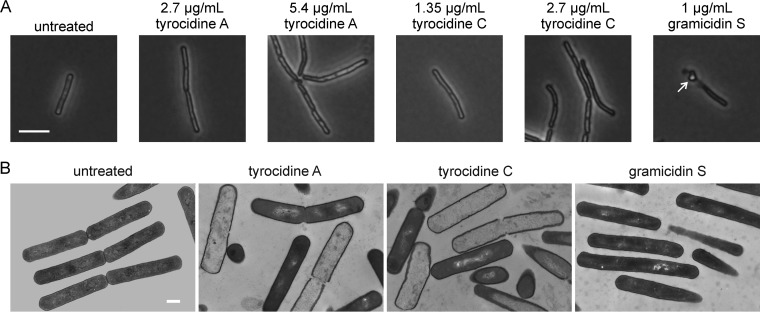
Effects of tyrocidines and gramicidin S on the cell wall. (A) Impact on cell wall integrity. B. subtilis 168 was treated with peptides for 10 min and subsequently fixed in a 1:3 mixture of acetic acid and methanol. This fixation method leads to extraction of the protoplast through holes in the peptidoglycan layer (arrow) when synthesis of the cell wall precursor lipid II is impaired (37, 45). Since this fixation method can sometimes lead to quick lysis of heavily damaged cells, we examined both 0.5× MIC (nonlytic) and 1× MIC of the tyrocidines but did not find membrane extrusions under both conditions. Bar, 2 µm. (B) Transmission electron microscopy pictures of B. subtilis 168 treated with 1× MIC of the peptides for 10 min. Bar, 0.5 µm.

To further examine the effects on cell wall integrity, we employed an organic fixation method that is indicative of holes in the peptidoglycan layer, typically related to impaired synthesis of the cell wall precursor lipid II (45). In line with an earlier study (39), gramicidin S clearly affected cell wall integrity as seen from the protoplast protruding through cell wall breaches (Fig. 2A). However, this phenotype was not observed for tyrocidines A and C, suggesting a clear mechanistic difference from gramicidin S. In fact, transmission electron microscopy revealed that the tyrocidines caused severe cellular damage, intracellular content leakage, and lysis of cells, whereas gramicidin S-treated cells showed only subtle cell shape alterations but no major signs of lysis or physical cell damage (Fig. 2B).

### Membrane permeability.

**FIG 3:**
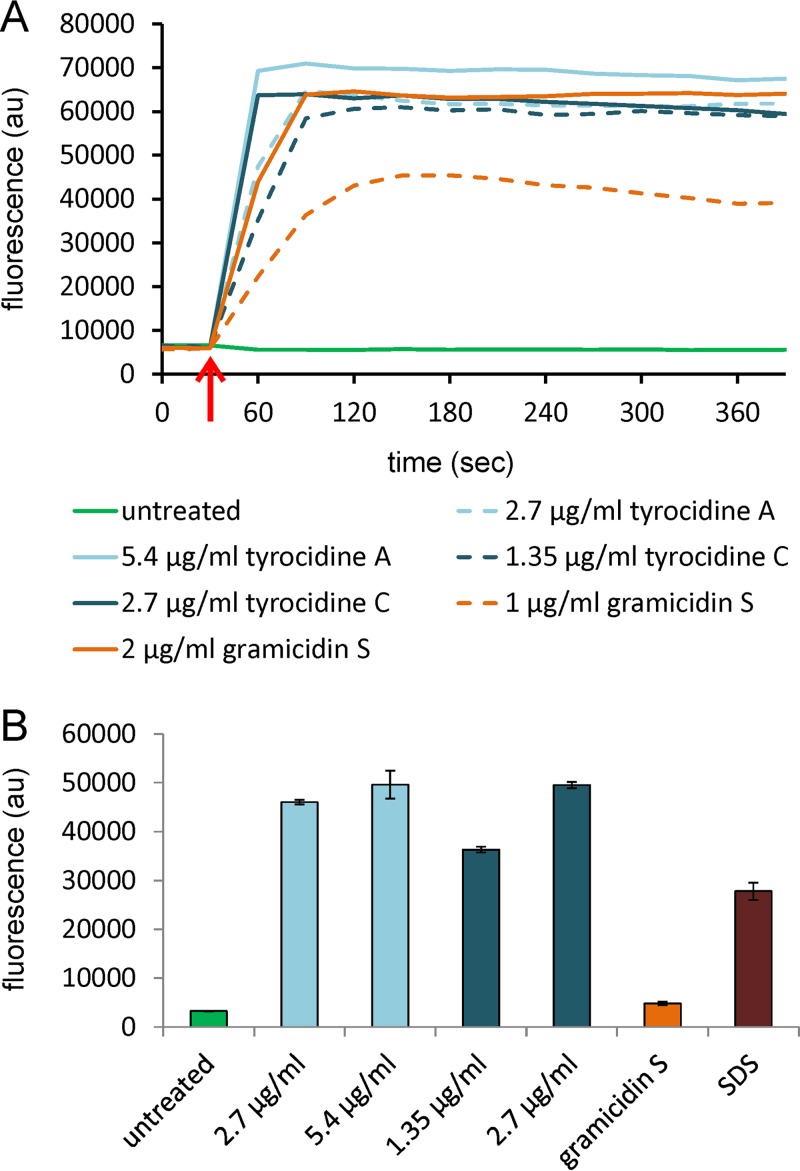
Tyrocidines but not gramicidin S form membrane pores *in vivo*. (A) Membrane potential measurements of exponentially growing B. subtilis 168 cells using the membrane potential-sensitive fluorescent probe DiSC(3)5. Tyrocidines A and C were applied at 0.5× and 1× MIC, and gramicidin S was applied at 1× and 2× MIC. The red arrow indicates the time point of antibiotic addition. (B) Membrane permeability for propidium iodide. Exponentially growing cells were treated with peptides for 5 min. For the tyrocidines, 0.5× MIC (nonlytic) and 1× MIC were used; gramicidin S was applied at 2× MIC (2 µg/ml, lytic concentration). SDS at 0.01% served as a positive control. Strain, B. subtilis 168. au, arbitrary units.

The current belief is that both the tyrocidines and gramicidin S form pores in the cell membrane (26, 31, 33–37). However, for the tyrocidines this has actually never been shown in bacteria, and the pore-forming ability of gramicidin S is heavily disputed (31, 33–37). Therefore, we examined the effects of these peptides on membrane permeability in detail. First, we determined the effects of the peptides on the membrane potential using the membrane potentiometric dye DiSC(3)5. Tyrocidines A and C led to immediate strong depolarization of B. subtilis cells already at 0.5× MIC (Fig. 3A). Inhibitory concentrations of gramicidin S (1× MIC) only gradually and partially depolarized B. subtilis cells. Full depolarization was achieved only at 2× MIC, a concentration causing cell lysis (Fig. S1). These results suggest that the tyrocidines form membrane pores while gramicidin S does not.

**FIG 4:**
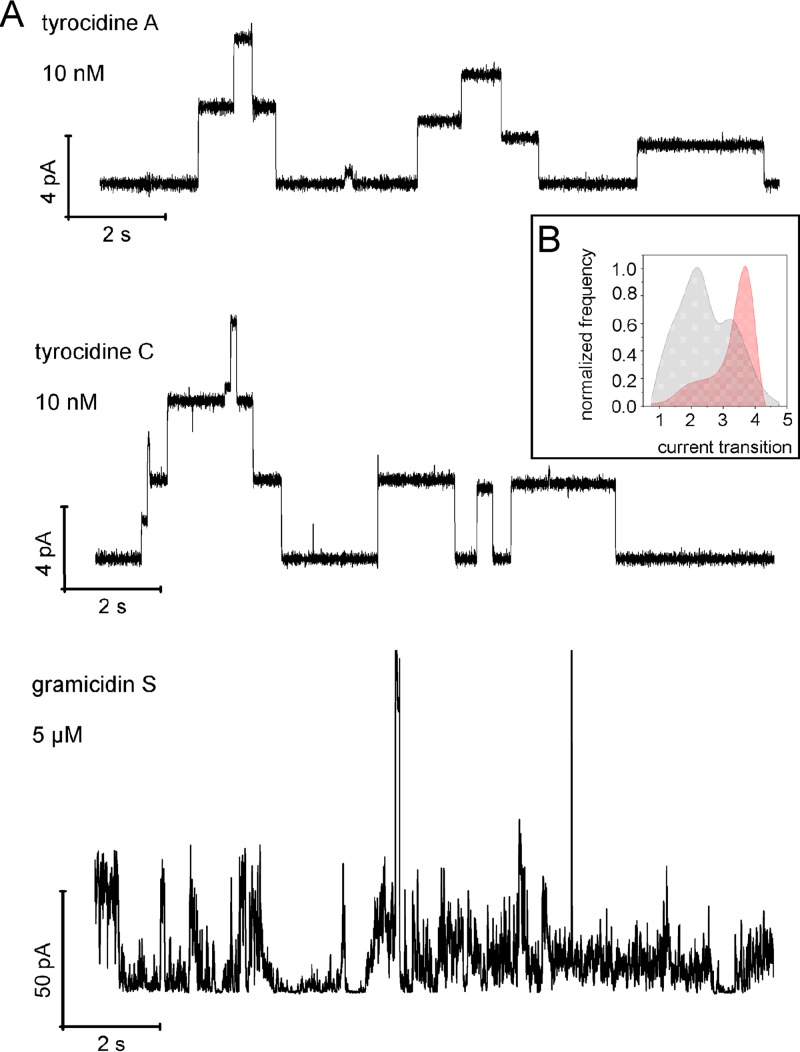
Conductivity measurements in model membranes. (A) Conductivity measurements in POPG-POPE (3:1) planar model membranes with an artificial transmembrane potential of 100 mV. Representative recordings of single-channel currents of tyrocidine A and tyrocidine C are shown. No ion conductance events were observed for gramicidin S up to concentrations leading to membrane collapse (5 µM). Note the different peptide concentrations and the different *y* scales. (B) Single-channel current transition amplitude histograms of tyrocidine A (gray) and tyrocidine C (red) in POPG-POPE (3:1) membranes recorded at 100 mV. Normalized frequency refers to the number of times that a pore with a specific current amplitude was observed and is displayed as a fraction of the maximum count. Data from 8 independent membranes and 158 single-channel transitions for tyrocidine A and 12 independent membranes with 560 single-channel transitions for tyrocidine C are shown.

To further characterize the ability of the peptides to generate distinct ion-conducting pores, we performed *in vitro* conductivity measurements. This allowed us to follow single-ion current events in a planar model lipid system mimicking Gram-positive membranes (3:1 mixture of 1-palmitoyl-2-oleoyl-*sn*-glycero-3-phosphoglycerol [POPG] and 1-palmitoyl-2-oleoyl-*sn*-glycero-3-phosphoethanolamine [POPE]). In line with our *in vivo* data, we observed a number of conductivity events for tyrocidines A and C at low, nanomolar concentrations (Fig. 4A). Current transitions peaked at 2 pA for tyrocidine A and 3.5 pA for tyrocidine C, corresponding to calculated conductance values of 20 pS and 35 pS, respectively (Fig. 4B). These conductance events are representative of several single-membrane pores that stay open for up to several seconds. Conductance events induced by gramicidin S were observed only at concentrations leading to membrane collapse (5 µM, Fig. 4A), lasted only milliseconds, and exhibited a broad range of amplitudes (Fig. 4A), which is indicative of general bilayer distortion. Similar behavior has been reported for gramicidin S in diphytanoyl phospholipid bilayers (31). These results show that both tyrocidine A and tyrocidine C induce discrete long-lived ion-conducting pores, while gramicidin S did not exhibit pore-forming capacity in our system.

To gain insight into whether tyrocidine pores are small ion channels or large membrane pores that allow big organic molecules to pass through, pore formation was assessed by following the influx of the fluorescent dye propidium iodide, which enters bacterial cells only through membrane lesions large enough for the bulky molecule to pass (46). Both tyrocidine A and tyrocidine C rapidly formed large pores in B. subtilis cells (Fig. 3B). Cells treated with gramicidin S did not show any propidium iodide influx, not even at bacteriolytic concentrations (2× MIC) (Fig. 3B; Fig. S1).

Taken together, both *in vivo* (Fig. 1 to 3) and *in vitro* (Fig. 4) experiments strongly indicated that at inhibitory concentrations tyrocidines A and C, but not gramicidin S, form large, ion-conducting pores. The slow and incomplete depolarization by gramicidin S (Fig. 3A) suggests rather a limited increase of passive membrane permeability.

### Membrane protein localization.

**FIG 5:**
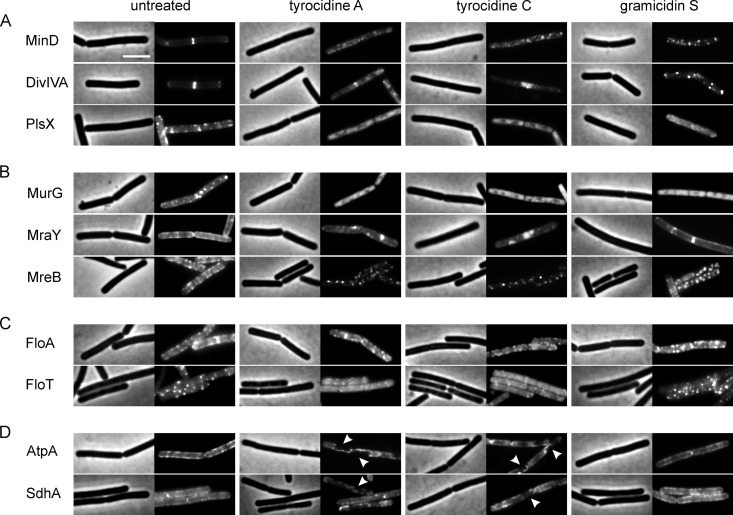
Delocalization of membrane proteins labeled with GFP. (A) Reporter proteins for membrane depolarization. Membrane localization of MinD and DivIVA requires the membrane potential, whereas membrane binding of the peripheral membrane protein PlsX is independent of the membrane potential. (B) Localization of proteins associated with cell wall synthesis. In contrast to MreB, localization of the peripheral MurG and integral MraY proteins is not reliant on the membrane potential. (C) Localization of flotillins involved in the organization of lipid rafts. FloA is an integral membrane protein, and FloT is a peripheral membrane-associated protein. (D) Localization of proteins involved in membrane-bound energy generation. AtpA and SdhA are the peripheral subunits of the ATP synthase and succinate dehydrogenase, respectively. Arrowheads indicate regions from which AtpA and SdhA are fully excluded. B. subtilis strains were grown in LB with appropriate inducer concentrations (see Table S1) and treated with peptides in early exponential growth phase for 10 min. Bar, 2 µm.

At MICs, gramicidin S did not fully depolarize the cell membrane (Fig. 3A) and even at supra-MICs, it did not lead to large membrane lesions (Fig. 3B). It is therefore plausible that other vital processes must be disturbed that, together with limited depolarization and impairment of the cell wall (Fig. 2A and 3A), will eventually lead to cell death. It is also well known that the membrane potential is required for the localization of peripheral membrane proteins involved in cell division and cell morphology (47), and pore formation by the tyrocidines is likely to affect different essential cellular processes. To investigate whether insertion of gramicidin S and the tyrocidines affects membrane proteins differently, we examined the localization of fluorescent membrane protein fusions involved in a variety of cellular processes. Figure 5A shows peripheral membrane proteins that can be used as reporters for dissipation of the membrane potential. The cell division proteins MinD and DivIVA require the membrane potential for their localization at the membrane (44, 47), while localization of the phospholipid synthase PlsX is independent of the proton motive force (44). In line with membrane depolarization, all three peptides delocalized MinD and DivIVA, whereby the tyrocidines caused a more diffuse cytoplasmic fluorescence signal, while gramicidin S induced stronger clustering of the proteins at the cell periphery (Fig. 5A). Interestingly, the phospholipid synthase PlsX completely lost its membrane localization after treatment with both the tyrocidines and gramicidin S. Since PlsX does not depend on the membrane potential, its delocalization demonstrates that the peptides have other effects on the membrane than only interfering with the barrier function of the cell membrane.

10.1128/mBio.00802-18.10 TABLE S1 B. subtilis strains used in this study. *gfp*, green fluorescent protein; *mgfp*, monomeric *gfp*; *sfgfp*, superfolder *gfp*; *msfgfp*, monomeric superfolder *gfp*; *yfp*, yellow fluorescent protein. Download Table S1, DOCX file, 0.0 MB. Copyright © 2018 Wenzel et al. 2018 Wenzel et al. This content is distributed under the terms of the Creative Commons Attribution 4.0 International license.

The cell wall-damaging effect of gramicidin S has been attributed to detachment of the peripheral peptidoglycan synthesis enzyme MurG, involved in the last steps of lipid II synthesis (39). To test whether the localization of this protein and other cell wall synthesis proteins is affected by the tyrocidines, we examined the localization of MurG, the transmembrane lipid I synthase MraY, and the actin homologue MreB, which coordinates lateral peptidoglycan synthesis (48, 49). As shown in Fig. 5B, MurG completely lost its membrane localization after treatment with all three peptides. However, MraY was clustered into huge foci by tyrocidines A and C, while gramicidin S did not show a clear effect. MreB is known to delocalize after membrane depolarization (47, 50). After treatment with the tyrocidines and, to a lesser extent, also with gramicidin S, MreB localized into discrete clusters distributed all over the cell membrane (Fig. 5B), a peculiar effect not seen before with membrane-targeting antibiotics (44, 47, 50).

The actin homologue MreB has been implicated in the formation of fluid membrane microdomains, so-called regions of increased fluidity (RIFs), which play a role in spatial organization of cell envelope synthesis (44, 50, 51). This made us curious whether the peptides would also affect the distribution of bacterial flotillin proteins involved in the formation of specific rigid membrane domains (known as lipid rafts in eukaryotes) (52, 53). While the integral flotillin FloA was not affected by any of the peptides, the peripheral flotillin FloT was clearly detached from the membrane by tyrocidines A and C but not by gramicidin S (Fig. 5C). FloT interacts with many membrane-associated proteins (54), and the separation from its interaction partners by dissociation from the membrane is likely to affect multiple cellular processes, including membrane organization, fluidity, respiration, protein secretion, membrane transport, signal transduction, and autolysin activity (53, 56–58).

Since gramicidin S was shown to reduce ATP levels in B. subtilis (39) and tyrocidines reduced oxygen consumption in S. aureus (28), we also examined their effects on the localization of AtpA, the peripheral subunit of the ATP synthase, and SdhA, the flavoprotein subunit of the succinate dehydrogenase (complex II) of the respiratory chain. These transmembrane protein complexes are unaffected by membrane potential-dissipating drugs like carbonyl cyanide *m*-chlorophenylhydrazone (CCCP) and valinomycin, and also the membrane-targeting antibiotic daptomycin (44, 47). Surprisingly, both AtpA and SdhA partially lost their membrane association after treatment with the tyrocidines (Fig. 5D), explaining inhibition of electron transport chain enzymes reported in an early study (29, 59). In contrast, gramicidin S had no marked effect on the localization of these proteins, suggesting that its effect on cellular ATP levels is probably due to delocalization of cytochrome *c* (39) and partial membrane depolarization.

### Gel-phase domains.

**FIG 6:**
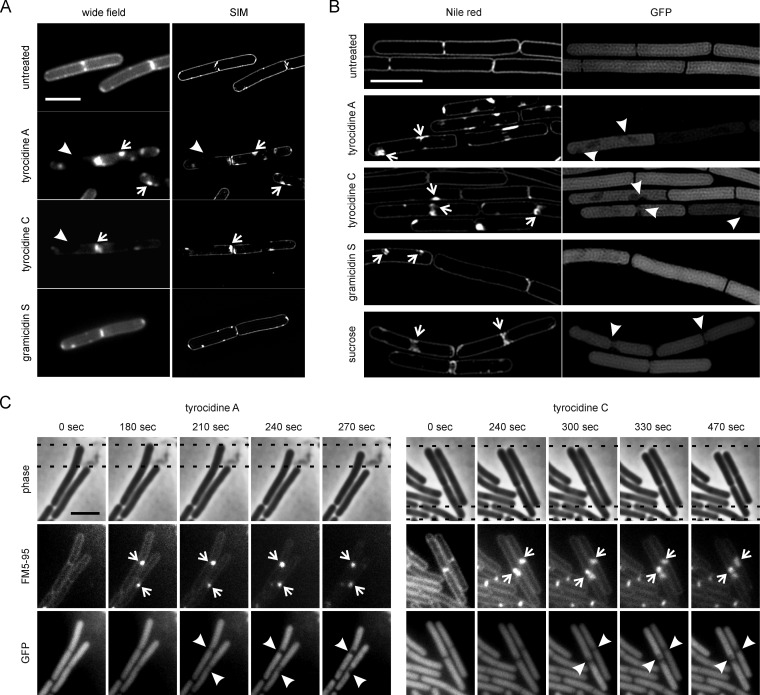
Tyrocidines and gramicidin S cause aberrant membrane staining and plasmolysis. (A) SIM images of B. subtilis 168 stained with MitoTracker green. Note the unstained membrane areas (arrowheads) and bright lipid accumulations (arrows) in cells treated with tyrocidines A and C. See also Fig. S5A for quantification of membrane patches. (B) Tyrocidines induce membrane invaginations. SIM images of B. subtilis bSS82 expressing cytosolic GFP from the strong ribosomal PrpsD promoter were stained with Nile red. Sucrose (20%) was used to induce osmotic upshock and used as a control for plasmolysis (bottom panel). (C) Tyrocidines cause plasmolysis. Time-lapse images of B. subtilis bSS82 cells treated with tyrocidines A and C. Membranes were stained with the nontoxic membrane dye FM5-95. Arrows indicate membrane patches and corresponding areas with reduced intracellular GFP. Bars, 2 µm. Experiments were performed in early exponential log phase using 1× MIC of the peptides.

Interestingly, many of the tyrocidine-treated cells displayed large areas from which AtpA and SdhA were excluded (Fig. 5D, arrowheads; Fig. S2, left panels; and Fig. S3A). It seems as if these empty areas might have been caused by plasmolysis of the cell membrane due to partial release of cytoplasmic content. However, the corresponding phase-contrast images did not indicate that these were partially empty cells (Fig. 5D and Fig. S2, right panels). To examine the effects of the peptides on the cell membrane in more detail, we employed superresolution structured illumination microscopy (SIM) and stained the cell membrane with MitoTracker green, a very bright fluorescent membrane stain that provides excellent SIM contrast (60). As shown in Fig. 6A, a 10-min incubation with the tyrocidines resulted in strongly fluorescent foci (open arrows) but also regions that were completely unstained (closed arrows). Gramicidin S-treated cells did not cause gaps in the fluorescent membrane stain but some small fluorescent membrane foci. When we expressed green fluorescent protein (GFP), it became apparent that many of the tyrocidine-treated cells lost their cytosolic GFP signal (Fig. 6B), indicating leakage of cellular content. Cells were counterstained with the red fluorescent membrane dye Nile red, which showed similar gaps in its fluorescent stain (Fig. 6B) as observed with MitoTracker green (Fig. 6A). Importantly, these gaps were also observed in cells that still contained their cytoplasmic GFP (Fig. 6B and Fig. S3B), indicating that these gaps are not caused by the absence of cell membrane due to lysis. The only remaining explanation for the absence of both fluorescent membrane dyes as well as membrane proteins is that these membrane areas are in a rigid, gel-phase state that excludes most dyes and proteins (61). Although stabilization of gel-phase domains by antimicrobial peptides has been observed *in vitro* (62, 63), to the best of our knowledge, such domains have not been documented before in bacterial cells.

10.1128/mBio.00802-18.4 FIG S2 Overview pictures of B. subtilis BS23 expressing AtpA-GFP. Cells were treated with 1× MIC of the peptides in early logarithmic growth phase. (Left panels) GFP channel. (Right panels) Phase-contrast channel. Bar, 2 µm. Download FIG S2, TIF file, 0.6 MB. Copyright © 2018 Wenzel et al. 2018 Wenzel et al. This content is distributed under the terms of the Creative Commons Attribution 4.0 International license.

10.1128/mBio.00802-18.5 FIG S3 Quantification of membrane domains. (A) Quantification of gel-phase domains. B. subtilis BS23 cells expressing AtpA-GFP were treated with peptides in early logarithmic growth phase. A minimum of 50 cells were examined per condition. (B) Unstained membrane areas are not representative of destroyed membranes. SIM pictures of B. subtilis bSS82 expressing intracellular GFP and stained with Nile red are shown. Arrows indicate unstained membrane domains in cells retaining intracellular GFP, showing that unstained membrane domains are not representative of destroyed membranes. Cells were grown until early exponential growth phase and treated with 1× MIC of tyrocidines A and C, respectively. Pictures were taken after 10 min of treatment. Bar, 2 µm. (C) Quantification of fluid membrane patches. B. subtilis 168 was grown until early exponential growth phase and treated with 1× MIC of tyrocidines A and C, respectively. Membranes were stained with FM5-95. A minimum of 300 cells were examined per condition. (D) Formation of fluid membrane patches is independent of protein and lipid synthesis. B. subtilis 168 was treated with inhibitory concentrations of either chloramphenicol (100 µg/ml) or triclosan (2.5 µg/ml) for 10 min to inhibit synthesis of proteins or lipids, respectively. Cells were then treated with peptides for an additional 10 min. Membranes were stained with FM5-95. Arrows indicate membrane patches. Download FIG S3, TIF file, 1.6 MB. Copyright © 2018 Wenzel et al. 2018 Wenzel et al. This content is distributed under the terms of the Creative Commons Attribution 4.0 International license.

### Accumulation of membrane material.

Cells stained with membrane dyes and treated with gramicidin S, and especially with tyrocidines A and C, showed membrane patches that were highly fluorescent (Fig. 6A and B and Fig. S3B and C). In the case of the tyrocidines, these membrane patches seemed to partially displace the cytoplasmic GFP signal (Fig. 6B, arrowheads), suggesting that the increased fluorescence signal is caused by an accumulation of membrane material (44, 50, 64). Formation of this extra membrane material can be caused by overactivation of fatty acid synthesis (65). However, when either fatty acid synthesis or protein synthesis was blocked with triclosan or chloramphenicol, respectively, bright membrane patches were still observed upon peptide treatment (Fig. S3D). Alternatively, these membrane accumulations can be caused by plasmolysis due to a loss of turgor pressure and cell shrinkage, as shown by plunging cells in a sucrose solution (Fig. 6B, bottom panel) (66). To confirm that tyrocidines A and C cause cell shrinkage, we performed a time-lapse experiment using cells stained with the membrane dye FM5-95. As shown in Fig. 6C, membrane patches are formed within minutes, rapidly followed by reduction of the GFP signal in their immediate proximity and cell shrinkage. Thus, tyrocidines induce large fluorescent membrane invaginations (patches) by causing a loss of turgor and subsequent cell shrinkage.

### Impact on membrane fluidity.

**FIG 7:**
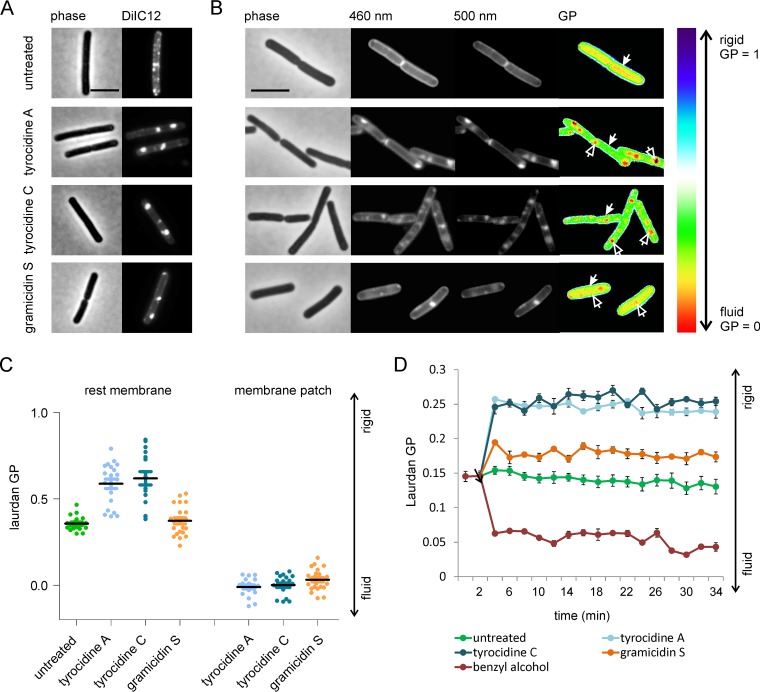
Effect of tyrocidines and gramicidin S on membrane fluidity. (A) Fluid lipid domain (RIF) staining with DiIC12. (B) Laurdan GP microscopy indicating local fluidity differences. Filled arrows indicate the cell membrane; open arrows indicate fluid membrane patches. (C) Quantification of membrane fluidity from laurdan microscopy pictures. The minimum GP within the membrane patch and the average GP in the lateral membrane were measured in individual cells. (D) Changes in overall membrane fluidity measured by laurdan GP in microtiter plate cultures. The membrane fluidizer benzyl alcohol served as a positive control. The arrow indicates the time point of antibiotic addition. Error bars represent standard deviations of the mean of three replicate experiments.

No signs of plasmolysis were observed with gramicidin S (Fig. 6B), in line with the fact that this antibiotic does not cause rapid cell lysis at inhibitory concentrations (Fig. 1D). However, gramicidin S also caused highly fluorescent membrane patches. Irregular membrane staining of fluorescent membrane dyes has been observed before when cells were treated with the membrane antibiotic daptomycin and the proton ionophore CCCP, and the irregularly stained areas were identified as highly fluid membrane domains, which increase the fluorescence of membrane dyes and can also accommodate more dye (50, 64). To examine whether these bright fluorescent membrane patches are fluid lipid domains, we used the membrane dye DiIC12, which displays a strong affinity for fluid membranes, including RIFs, due to its short hydrocarbon tail (50, 67, 68). Indeed, after treatment with gramicidin S the natural distribution of RIFs was severely disturbed, and the dye accumulated in one or two large patches per cell (Fig. 7A). The same patches were observed in cells treated with the tyrocidines (Fig. 7A), and they seem to correspond to the large membrane invaginations caused by plasmolysis (Fig. 6). Presumably, fluid membrane domains are more likely to invaginate due to the higher flexibility of fluid lipids.

To confirm that these large DiIC12-stained membrane patches are indeed more fluid, we employed laurdan generalized polarization (GP). The fluorescence membrane dye laurdan shifts its fluorescence spectrum depending on the amount of water molecules in the bilayer, and laurdan GP can therefore be used as a measure of phospholipid head group spreading and fatty acid chain flexibility (69, 70). As shown in Fig. 7B, this assay confirmed that the membrane patches (open arrows) have an increased fluidity compared to the bulk membrane (filled arrows). When we quantified laurdan GP in the patches and the remaining membrane, it became apparent that not only did the peptides cause fluid membrane patches but this also led to a rigidification of the rest of the membrane (Fig. 7C).

To gain insight into how fast these fluidity changes happen, laurdan GP was measured spectroscopically in batch culture. We found that there was a rapid (2-min) reduction of membrane fluidity when the peptide antibiotics were added (Fig. 7D), indicating that this is a direct effect of peptide-membrane interaction and not a bacterial adaptation strategy, which would require the induction of specific enzymes and thus take more time. In line with our laurdan microscopy data, the overall membrane rigidification was stronger for tyrocidines A and C than for gramicidin S (Fig. 7D), supporting our notion that the tyrocidines change the physical parameters of the lipid bilayer in a different way than gramicidin S.

### Protein delocalization is independent of MreB.

**FIG 8:**
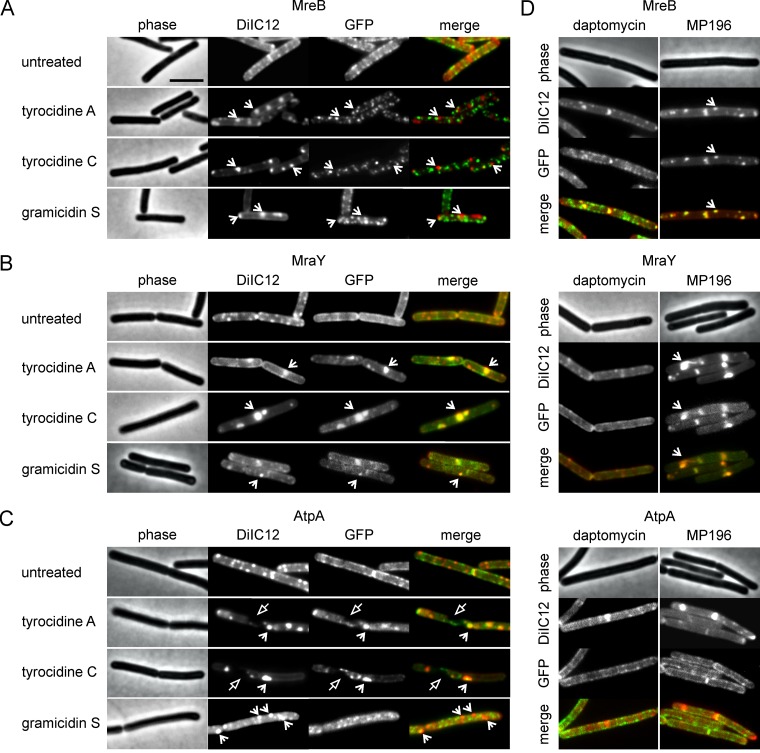
Overlap of GFP-tagged proteins with fluid membrane domains. (A to C) Colocalization of MreB (A), MraY (B), and AtpA (C) with DiIC12 was tested after a 10-min treatment with tyrocidine A, tyrocidine C, and gramicidin S. Open arrows indicate fluid membrane patches induced by the peptides. Closed arrows indicate membrane areas from which both DiIC12 and AtpA are excluded. (D) Effects of the unrelated antimicrobial peptides daptomycin and MP196. Cells were treated with 1 µg/ml daptomycin (in LB supplemented with 1.25 mM CaCl_2_) or 10 µg/ml MP196. B. subtilis strains were grown in LB supplemented with appropriate inducer concentrations (Table S1) and treated with peptides in early exponential growth phase for 10 min. Bar, 2 µm.

Previous work suggested that RIFs are formed by MreB polymers, both in B. subtilis and in E. coli (50, 51). All three peptides caused clustering of MreB (Fig. 5B); therefore, we examined whether these MreB clusters are responsible for the large DiIC12-stained RIFs that emerge upon addition of the peptide antibiotics (Fig. 7A). Interestingly, the GFP-MreB clusters did not overlap DiIC12 clusters, both in the case of gramicidin S and in the case of the tyrocidines (Fig. 8A). Thus, the peptide antibiotics are able to form artificial RIFs without the help of MreB. To confirm this, we tested a triple deletion strain lacking MreB and its homologues MreBH and Mbl. Indeed, in such mutant cells the addition of gramicidin S and the tyrocidines still resulted in large DiIC12-stained membrane patches (Fig. S4).

10.1128/mBio.00802-18.6 FIG S4 Fluid lipid clustering does not require MreB. (A) Lipid patches are independent of MreC (strain 3481), the membrane-anchoring protein of MreB. (B) Lipid patches are independent of MreB. MreB organizes RIFs in B. subtilis, and its delocalization results in their accumulation. Thus, membrane patches caused by delocalization of MreB or its homologues should be absent in the Δ*mreB* Δ*mbl* Δ*mreBH* mutant (strain 4277). Naturally occurring RIFs are indicated by filled arrows; fluid lipid accumulations (clustered RIFs) are indicated by open arrows. Bars, 2 µm. All experiments were performed in early exponential growth phase. Download FIG S4, TIF file, 0.6 MB. Copyright © 2018 Wenzel et al. 2018 Wenzel et al. This content is distributed under the terms of the Creative Commons Attribution 4.0 International license.

To our surprise, untreated MreB triple mutant cells also showed some small RIFs (Fig. S4). This is in disagreement with what we have reported previously (50). An in-depth analysis revealed that DiIC12-stained RIFs become visible only during logarithmic growth and disappear when cells enter the stationary phase of growth, despite the fact that MreB is still present in the stationary phase (Fig. S4 to S6 and Text S1). This growth-phase-dependent occurrence of RIFs explained the differences from our previous finding. Based on this and previous studies, we propose that MreB is not involved in establishing RIFs but rather helps to organize them along the cell axis.

10.1128/mBio.00802-18.1 TEXT S1 MreB is not required to establish RIFs. Download Text S1, DOCX file, 0.0 MB. Copyright © 2018 Wenzel et al. 2018 Wenzel et al. This content is distributed under the terms of the Creative Commons Attribution 4.0 International license.

10.1128/mBio.00802-18.7 FIG S5 Formation of RIFs depends on the growth phase and not on the presence of MreB. (A) Growth of B. subtilis 168 (wild type [WT], green) and B. subtilis 4277 (Δ*mreB* Δ*mreBH* Δ*mbl*, blue) in LB supplemented with 20 mM MgCl_2_ at 30°C. Green (strain 168) and blue (strain 4277) arrows and dashed lines represent time points used for DiIC12 microscopy in panel B. The red line represents the time point (150 min) used for DiIC12 microscopy in an earlier study that did not find RIFs in B. subtilis 4277 (50). At this time point, the WT has an OD of approximately 0.25 (exponential phase) while the triple *mreB* mutant has an OD of approximately 0.15 (lag phase). Time points at which microscopically visible RIFs were observed are marked with an asterisk. (B) DiIC12 microscopy of B. subtilis 168 (WT) and B. subtilis 4277 (Δ*mreB* Δ*mreBH* Δ*mbl*) at time points corresponding to the growth experiment shown in panel A. RIFs are only clearly visible during exponential growth phase in both strains. Note that, depending on the growth conditions (temperature, medium, and aeration), exponential growth phase can be reached at different incubation times and in a different OD range. Download FIG S5, TIF file, 1.2 MB. Copyright © 2018 Wenzel et al. 2018 Wenzel et al. This content is distributed under the terms of the Creative Commons Attribution 4.0 International license.

10.1128/mBio.00802-18.8 FIG S6 Growth of the Δ*mreC* mutant compared to the B. subtilis 168 WT and Δ*mreB* Δ*mreBH* Δ*mbl* mutant. The Δ*mreC* mutant grows slightly faster than the Δ*mreB* Δ*mreBH* Δ*mbl* strain, providing an explanation of why RIFs were observed with the Δ*mreC* strain but not with the Δ*mreB* Δ*mreBH* Δ*mbl* strain in our previous study (50). Cells were grown at 30°C in LB supplemented with 20 mM MgCl_2_. Download FIG S6, TIF file, 0.1 MB. Copyright © 2018 Wenzel et al. 2018 Wenzel et al. This content is distributed under the terms of the Creative Commons Attribution 4.0 International license.

### Peptides induce fluid lipid domains by stabilizing the liquid crystalline phase.

The previous results suggested that both tyrocidines and gramicidin S induce fluid lipid domains independently from the MreB cytoskeleton. To confirm that the observed fluidity changes are a direct cause of peptide-lipid interaction, we tested whether the peptides influence phase separation behavior of model lipid membranes using ^31^P solid-state nuclear magnetic resonance (NMR). As before, we used 3:1 POPG-POPE model membranes to mimic Gram-positive bacterial cell membranes. To exclude the possibility that the observed effects correspond to head group demixing rather than transition from liquid crystalline to gel phase, we additionally tested pure POPE bilayers. All three peptides stabilized the liquid crystalline (fluid) phase in both model membrane systems (Fig. S7). For the tyrocidines, similar effects have been described before in model membranes made of dielaidoylphosphatidylethanolamine (DEPE) (26), and gramicidin S has been shown to have a preference for more-fluid membranes (32). The NMR data indicate that the generation of fluid lipid domains is a direct cause of peptide-lipid interaction and suggest a model in which the peptides preferentially insert into fluid lipid domains like RIFs and increase their local fluidity. The DiIC12 and laurdan data suggest that these domains also attract more flexible (fluidizing) lipid species, further increasing local fluidization.

10.1128/mBio.00802-18.9 FIG S7 ^31^P solid-state NMR spectra of model bilayers. POPG-POPE (A) (at 3:1) or POPE (B) bilayers were mechanically oriented with the normal parallel to the magnetic field direction and equilibrated at 93% relative humidity in the absence and presence of 2% tyrocidine A, 2% tyrocidine C, and 2% gramicidin S at the indicated temperatures. In the absence of peptide, the ^31^P spectra exhibited a predominant peak at 30 ppm (310 K), indicative of a good alignment of the phospholipid head groups. This value shifted to 35 ppm below the phase transition temperature of the lipid mixture (290 K). At intermediate temperatures, two ^31^P NMR peaks are distinguishable, suggesting the coexistence of liquid crystalline and gel-phase lipid domains. The presence of tyrocidine A at a lipid-to-peptide ratio of 50 was found to have minimal effects on the bilayer orientation with only a small increase in the “nonoriented” spectral intensities extending up to −11 ppm. These are indicative of minor rearrangements at the level of the phospholipid head groups or due to loss of orientational order of lipid membrane domains. The transition to the gel phase was lowered by 5 to 10 K, suggesting that the peptide stabilizes the liquid crystalline phase. Whereas the spectral line boarding is similar in the presence of tyrocidine C and gramicidin S, the effect of these peptides on the phase transition temperature was less pronounced. The ^31^P solid-state NMR signals that arise from the liquid crystalline and gel phase were then integrated and compared to each other. The resulting contribution from the liquid crystalline signal is shown as a function of temperature for POPG-POPE (3:1) (C) and POPE (D) oriented bilayers. The presence of 2 mol% of tyrocidine A, tyrocidine C, or gramicidin S all shifts the phase transition to lower temperatures than the control. Download FIG S7, TIF file, 0.7 MB. Copyright © 2018 Wenzel et al. 2018 Wenzel et al. This content is distributed under the terms of the Creative Commons Attribution 4.0 International license.

### Membrane proteins localize to fluid domains.

The relatively bulky membrane-targeting domains of most peripheral membrane proteins need a certain flexibility of the lipid bilayer for their binding and insertion into the membrane (71). Similarly, the hydrophobic surface area of transmembrane proteins causes an inherent preference of these proteins for areas of a particular membrane thickness and fluidity (72). It is therefore likely that the segregation of lipids into fluid and rigid (membrane) domains by the tyrocidines and gramicidin S (Fig. 7B) is responsible for the observed delocalization of membrane proteins (Fig. 5). To investigate this, we examined the localization of MraY (transmembrane) and AtpA (peripheral subunit of a transmembrane complex) in relation to fluid lipid domains stained with DiIC12. MraY clearly overlapped DiIC12 clusters caused by the tyrocidines and gramicidin S, suggesting that this protein preferentially partitions from the rigidified membrane regions into fluid membrane patches (Fig. 8B). AtpA has been used as a reporter for cell membrane invagination (44, 50), and it does not accumulate in fluid lipid domains caused by antibiotics (50) (also MP196, Fig. 8B). In line with membrane invaginations due to plasmolysis caused by the tyrocidines but not by gramicidin S (Fig. 6), AtpA showed a substantial overlap with the tyrocidine-induced DiIC12 clusters but not with clusters induced by gramicidin S (Fig. 8C).

To compare the effects of the tyrocidines and gramicidin S with those of other membrane-targeting peptides, we performed the same costaining experiment with two well-studied antimicrobial peptides, the lipopeptide daptomycin, which selectively inserts into RIFs and tightly aggregates them into rigid clusters (44), and the small cationic peptide MP196, which disturbs membrane architecture and depolarizes the membrane by inhibiting the respiratory chain (39). In line with an earlier study (44), daptomycin only slightly affected the localization of MreB and had no effect on MraY and AtpA localization (Fig. 8B). On the other hand, MP196 caused MreB clusters that overlapped aggregated RIFs, which is typical for depolarizing compounds (50). MraY also accumulated in the DiIC12-stained membrane patches, whereas AtpA localization was unaffected. These effects are quite different from what was observed with the tyrocidines and gramicidin S, indicating that these old peptide antibiotics disrupt the bacterial cell membrane by thus-far unique mechanisms.

### Influence on the chromosome.

**FIG 9:**
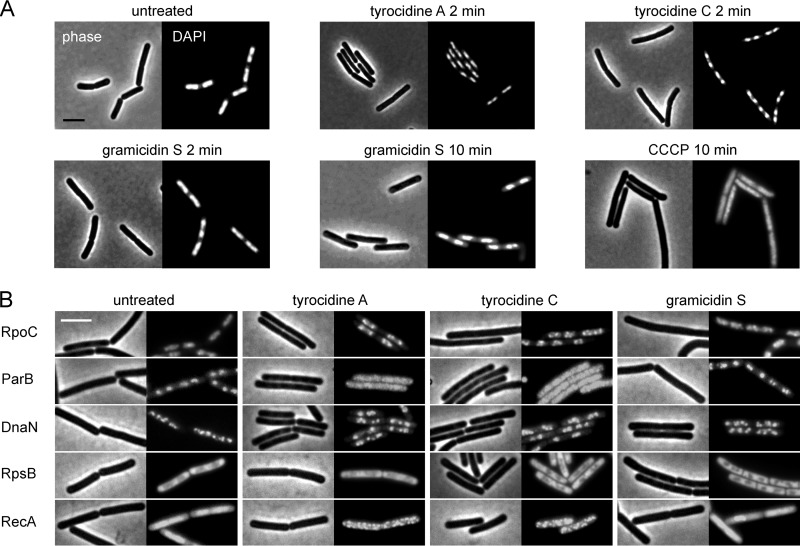
Influence of tyrocidines and gramicidin S on the bacterial chromosome. (A) Tyrocidines cause condensation of the nucleoid within 2 min. CCCP was included as a control for depolarization. DNA was stained with DAPI. Strain, B. subtilis 168. (B) Localization of DNA-associated proteins (see main text for details). B. subtilis strains were grown in LB with appropriate inducer concentrations (see Table S1) and treated with peptides in early exponential growth phase for 10 min. The ribosomal subunit protein RpsB was chosen as control for a cytosolic protein not associated with the nucleoid. Bars, 2 µm.

Although the bacterial cell membrane is considered the key target of both the tyrocidines and gramicidin S, *in vitro* experiments have shown that these antibiotics also interact with DNA (40–43). However, it has never been shown whether this DNA-binding activity plays a role *in vivo*. To investigate this, we stained the bacterial chromosome with the DNA-binding dye 4′,6-diamidino-2-phenylindole (DAPI). Cells treated with tyrocidines A and C showed a clear condensation of the nucleoid (Fig. 9A). As a control, we treated cells with CCCP but did not observe any nucleoid condensation, indicating that this is not a mere effect of membrane depolarization (Fig. 9A). Since nucleoid condensation occurred rapidly (<2 min) and coincided with shrinking of the cells, it is possible that loss of turgor pressure plays a role in tyrocidine-induced nucleoid condensation. In fact, an osmotic upshock can lead to plasmolysis, nucleoid compaction, and dissociation of RNA polymerase from the DNA (73). Therefore, we examined the localization of RNA polymerase by tagging its subunit RpoC with GFP. As shown in Fig. 8B, RNA polymerase remained attached to the nucleoid but seemed to cluster. To test whether the peptides influence DNA replication, we examined the localization of the DNA replication regulator ParB (Spo0J) (74, 75) and the DNA polymerase subunit DnaN. The ribosome subunit RpsB was included as a cytosolic control protein. Both tyrocidines caused a complete detachment of ParB from the origin of replication (Fig. 9B). DnaN remained attached to the chromosome but exhibited some clustering toward the center of the cells after treatment with tyrocidines A and C (Fig. 9B). The same effect is seen with the gyrase inhibitor ciprofloxacin (60), which causes arrest of the replication fork (76). RpsB, which is not associated with DNA, was not affected by the peptides. To examine whether the peptides cause DNA damage, we looked at the localization of RecA. Upon DNA damage, this repair protein forms large nucleoprotein filaments (77, 78). As shown in Fig. 8B, RecA formed clear foci after treatment with tyrocidines A and C. In contrast to the tyrocidines, gramicidin S had no effect on the localization of any of these proteins. These results indicate that the tyrocidines also target the bacterial DNA, resulting in inhibition of DNA replication and DNA damage, whereas gramicidin S does not show any DNA-targeting activity *in vivo*.

### Activity against persister cells.

**FIG 10:**
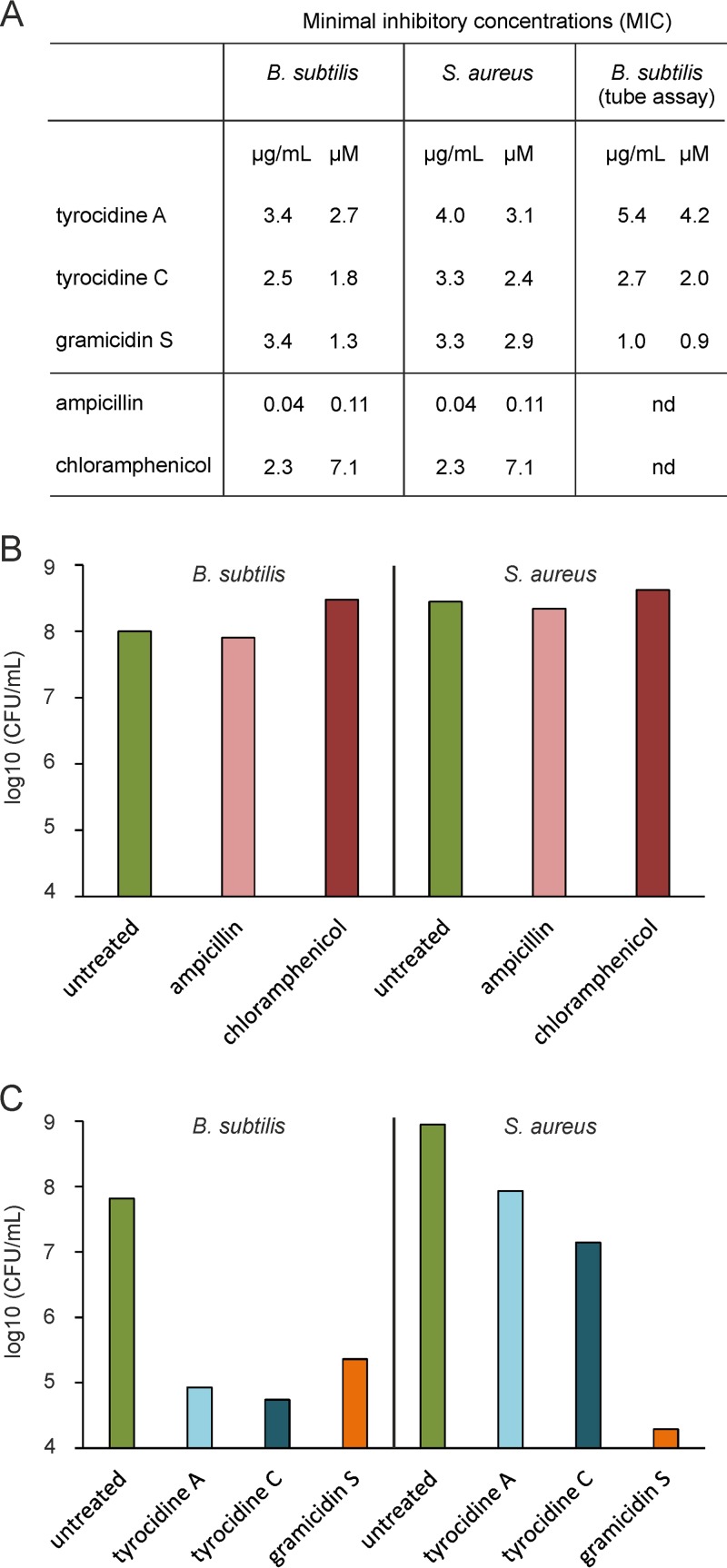
Activity against growing and nongrowing cultures of B. subtilis 168 and S. aureus 8325. (A) MIC(s) against B. subtilis, the model organism used in this study, and the pathogenic Gram-positive bacterium S. aureus. MICs for B. subtilis were additionally determined in a test tube assay to exactly match the conditions for mode-of-action experiments. (B) Log_10_ CFU reduction of nongrowing B. subtilis and S. aureus cultures treated with ampicillin and chloramphenicol. (C) Log_10_ CFU reduction of nongrowing B. subtilis and S. aureus cultures (stationary overnight cultures).

Antibiotic tolerance caused by nongrowing persister cells is an important problem in antibacterial therapy as it leads to chronic and recurrent bacterial infections (79). Membrane-active compounds have been suggested as treatment options for such persistent infections (80). Since the tyrocidines and gramicidin S affect multiple cellular processes, including the cell membrane, we tested the potential of the peptides to kill nongrowing overnight cultures of B. subtilis and S. aureus that have been used as simple model systems for persister cells (81, 82). As shown in Fig. 9A, ampicillin and chloramphenicol were highly active against growing B. subtilis and S. aureus cultures (Fig. 10A). However, overnight cultures were completely resistant to 9-h treatment with these antibiotics. In contrast, both the tyrocidines and gramicidin S were highly efficient in killing nongrowing B. subtilis cells (Fig. 10B). A 9-h incubation with the tyrocidines killed 90% to 99% of nongrowing S. aureus cells, whereas gramicidin S achieved a CFU reduction of as much as 5 log units (Fig. 10C), underlining the clinical potential of the peptides.

## DISCUSSION

There is a considerable amount of literature on how antimicrobial peptides interact with model membrane systems (26, 30, 31, 83, 84). However, only very limited information is available on how these antibiotics affect bacterial cells *in vivo*, even for tyrocidines and gramicidin S, the oldest natural peptide antibiotics in clinical use (2, 3). Here, we report the first comprehensive *in vivo* mode of action study of these cyclic β-sheet decapeptides and show that they act very differently, despite sharing 50% sequence homology.

Both the tyrocidines and gramicidin S affect membrane fluidity, membrane potential, and the localization of essential peripheral membrane proteins. However, the effect of tyrocidines on the cell membrane is much more severe, leading to membrane pores, phase separation into fluid membrane foci and rigid gel-phase domains, membrane invaginations, and delocalized transmembrane proteins.

We noticed only subtle differences between tyrocidines A and C. Tyrocidine C preferentially causes ion channels with higher current amplitudes, while tyrocidine A showed a broader distribution of current amplitudes in model membranes. Tyrocidine C possesses bulkier amino acid side chains (Trp-d-Trp) than tyrocidine A (Phe-d-Phe) at the variable aromatic dipeptide unit (Fig. 1A and B), which could indicate a stronger spreading of membrane lipids and therefore higher pore-forming capacity. In line with this, tyrocidine C was more active against B. subtilis and S. aureus than tyrocidine A, suggesting that pore formation is an important part of the mechanism of action of these peptides.

Gramicidin S appeared to work very differently than the tyrocidines. Gramicidin S does not form discrete membrane pores, affects only the localization of peripheral membrane proteins, and does not form overt gel-phase domains. However, like the tyrocidines, it does form large fluid membrane domains independent of MreB. Early *in vitro* studies have proposed lipid phase separation for the mechanism of gramicidin S (90), which is supported by our NMR results. The microscopic images of DiIC12-stained fluid domains show for the first time that lipid demixing does indeed occur *in vivo*.

It is curious that gramicidin S, which shares half of its sequence with the tyrocidines (Fig. 1A to C), has such different effects on the cell membrane. Both tyrocidines and gramicidin S insert superficially into the membrane, namely, at the interface between phospholipid head groups and fatty acid chains (32, 91, 92). However, these studies have been performed in different membrane systems, and at least for gramicidin S, it is known that affinity and penetration depth depend on both lipid head group composition and membrane fluidity (32). A systematic comparative study will be needed to reach a final conclusion on the differences in membrane interaction between these peptide antibiotics. Tyrocidines possess four aromatic residues, while gramicidin S has only two phenylalanine residues. Furthermore, both tyrocidines A and C form dimers, while dimerization of gramicidin S is deemed unlikely (91, 93). Therefore, one might speculate that the bulkier side chains of tyrocidines together with dimerization lead to stronger lipid spreading and, thus, more pronounced membrane effects than those of gramicidin S.

Fluid membrane domains are induced by all three peptides both *in vivo* and *in vitro*, showing that this kind of phase separation is not underlying pore formation. Instead, the formation of gel-phase domains and rigidification of the bulk membrane occurred only in cells treated with the pore-forming peptides tyrocidines A and C. The interface between membrane domains of considerably different fluidity constitutes a weak spot in the lipid bilayer and is assumed to facilitate ion leakage (85–89). It is likely that the interface between highly fluid membrane domains and the rigidified rest membrane plays a crucial role in the pore-forming ability of the tyrocidines. However, the exact mechanism of how the tyrocidines achieve membrane rigidification and induce gel-phase domains cannot be determined in this study.

Another important difference between gramicidin S and the tyrocidines is that the latter cause DNA damage and interfere with DNA-binding proteins. Tyrocidines contain two more aromatic amino acids than gramicidin S (Fig. 1A), and it has been shown that aromatic amino acids can intercalate between the N bases in DNA and interfere with N-base stacking interactions (94). Apparently, while gramicidin S is also able to bind to DNA (42) *in vitro*, this is not reflected in the *in vivo* situation.

So far, most antimicrobial peptides are studied in model membrane systems, resulting in pore formation being the prevailing mechanistic model for these compounds. However, biophysical studies are done with artificial membrane systems, conditions that fail to capture the complexity of biological membranes (44, 95). Our *in vivo* study sheds new light on the antibacterial mechanism of these pioneering cyclic β-sheet decapeptide antibiotics, revealing a much more multifaceted and complex mechanism than previously thought. This may explain why both the tyrocidines and gramicidin S are still so effective after decades of clinical application and why there is virtually no resistance to them. Interestingly, the effect of gramicidin S on the bacterial membrane is less severe than that of the tyrocidines, yet gramicidin S is equally as or even more efficient in killing both growing and nongrowing bacteria. The latter is interesting, since persistent and recurrent bacterial infections caused by nongrowing persister cells impose great difficulties in the clinic (79). While classical antibiotics such as chloramphenicol and ampicillin target biosynthetic processes, they fail to kill nongrowing cells. Therefore, membrane-active antibiotics have been proposed as treatment options for persistent infections (18, 79, 80). Our data on the tyrocidines and gramicidin S corroborate this. Since the tyrocidines impair the function of the bacterial membrane at both biosynthetic (cell division, cell wall, membrane, and ATP synthesis) and structural (flotillin, membrane phase separation, and membrane pores) levels, and additionally affect DNA packing, cells have little possibility to recover from exposure to these peptide antibiotics. Gramicidin S has less effect on the cell membrane and no effect on the bacterial nucleoid, yet it is only slightly less efficient against stationary B. subtilis and much more efficient against stationary S. aureus cells. S. aureus has a considerably different lipid composition than B. subtilis in terms of both head groups and fatty acids (96, 97), which might explain these differences. Our results underline that classic pore formation is not necessarily a requirement for efficient killing of bacteria.

## MATERIALS AND METHODS

Details on antibiotics, bacterial strains, and experimental procedures can be found in Text S2 in the supplemental material. A list of B. subtilis strains used in this study is displayed in Table S1. All strains were grown at 30°C under steady agitation in Luria-Bertani broth (LB). MICs were determined in a standard serial dilution assay. Growth experiments were carried out in 96-well format in a temperature-controlled BioTek Synergy MX plate reader under continuous shaking. All mode-of-action assays were performed with log-phase B. subtilis cells at an OD at 600 nm (OD_600_) of 0.3 at 30°C under steady agitation. Unless otherwise noted, cells were treated with 1× MIC of the respective antibiotics for 10 min. Fluorescence microscopy and staining of cells with fluorescent dyes were carried out as described previously (44, 60, 98). Electron microscopy was performed using a recently described flat embedding technique (60). The membrane potential was measured with DiSC(3)5 as described by Te Winkel et al. (98). Propidium iodide influx and laurdan spectroscopic assays were essentially performed as described by Müller et al. (44). Electrophysiological and NMR measurements were performed in 3:1 POPG-POPE or pure POPE, respectively, as described in Text S2. Time-lapse microscopy and SIM were essentially performed as described by Saeloh et al. (60). Activity against stationary-phase cells was determined using overnight cultures of B. subtilis and S. aureus. Antibiotic concentrations were adjusted to the higher cell count, and cells were incubated with antibiotics for 9 h prior to CFU determination.

10.1128/mBio.00802-18.2 TEXT S2 Supplemental materials and methods. Download Text S2, DOCX file, 0.0 MB. Copyright © 2018 Wenzel et al. 2018 Wenzel et al. This content is distributed under the terms of the Creative Commons Attribution 4.0 International license.
