# Supplementary figures and images for: The Multifaceted Antibacterial Mechanisms of the Pioneering Peptide Antibiotics Tyrocidine and Gramicidin S
Source: mBio. 2018 Oct 9;9(5):e00802-18. doi: 10.1128/mBio.00802-18 (PMC6178620; doi:10.1128/mBio.00802-18)

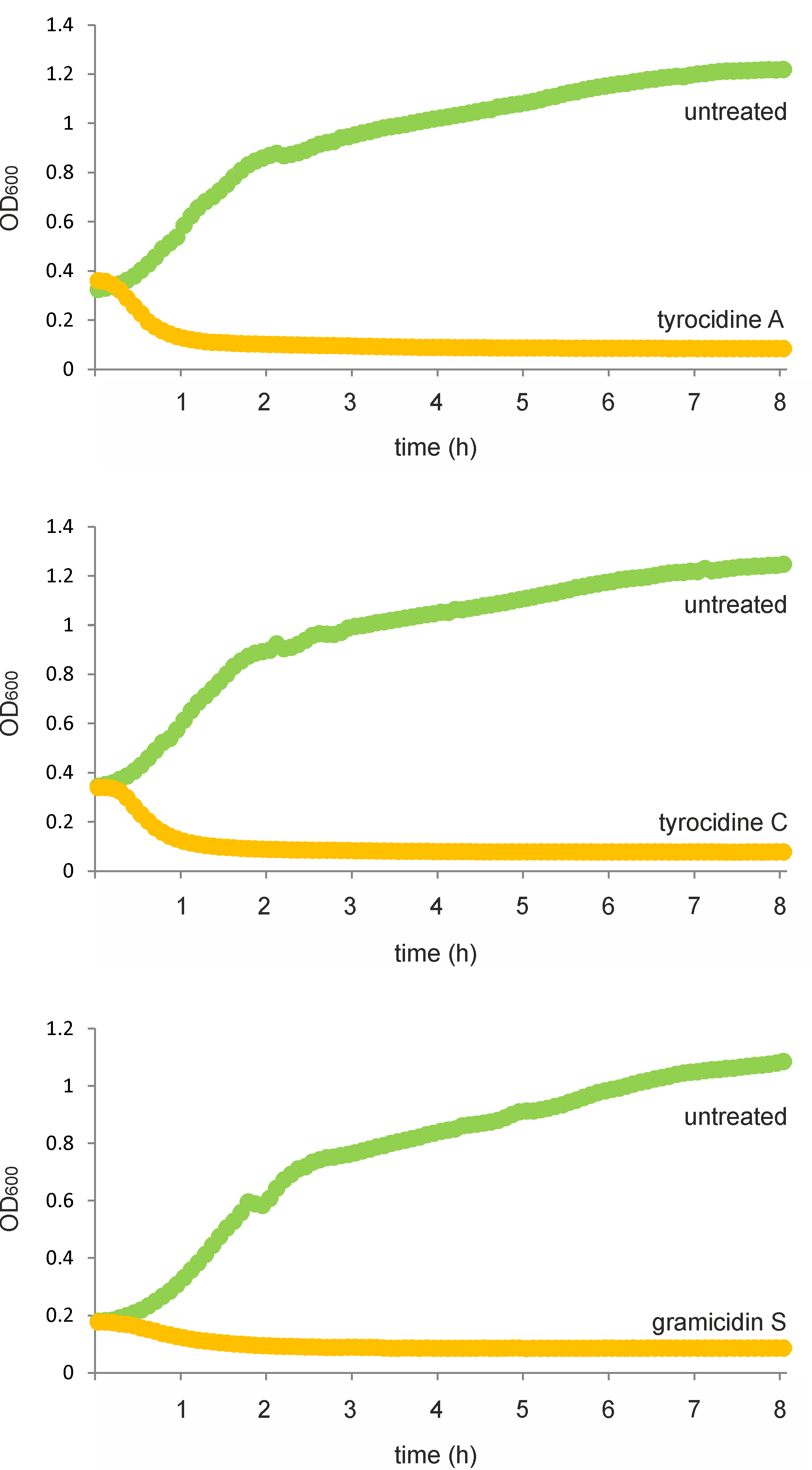

Supplement: FIG S1 [file mbo005184098sf1.tif]

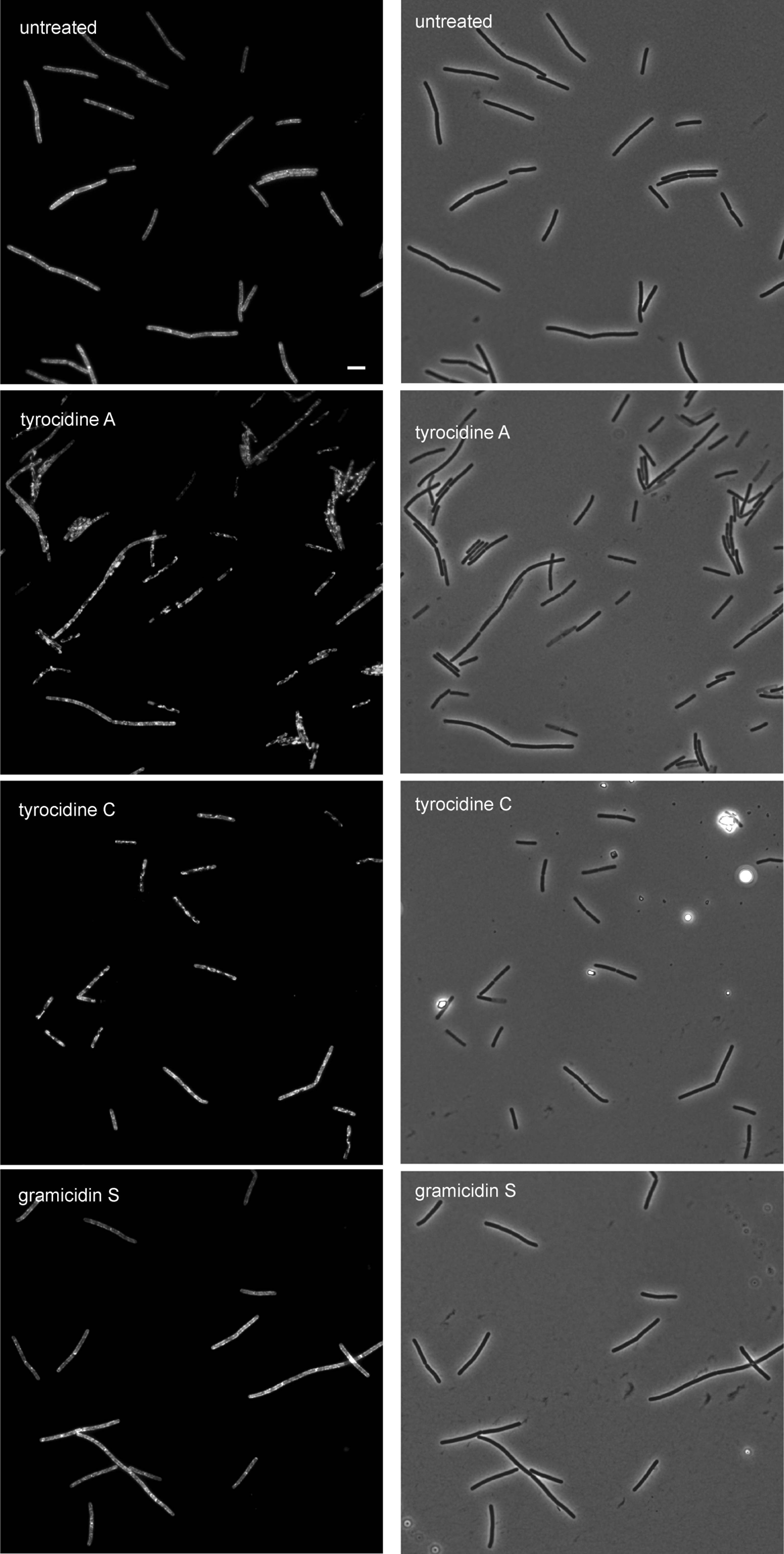

Supplement: FIG S2 [file mbo005184098sf2.tif]

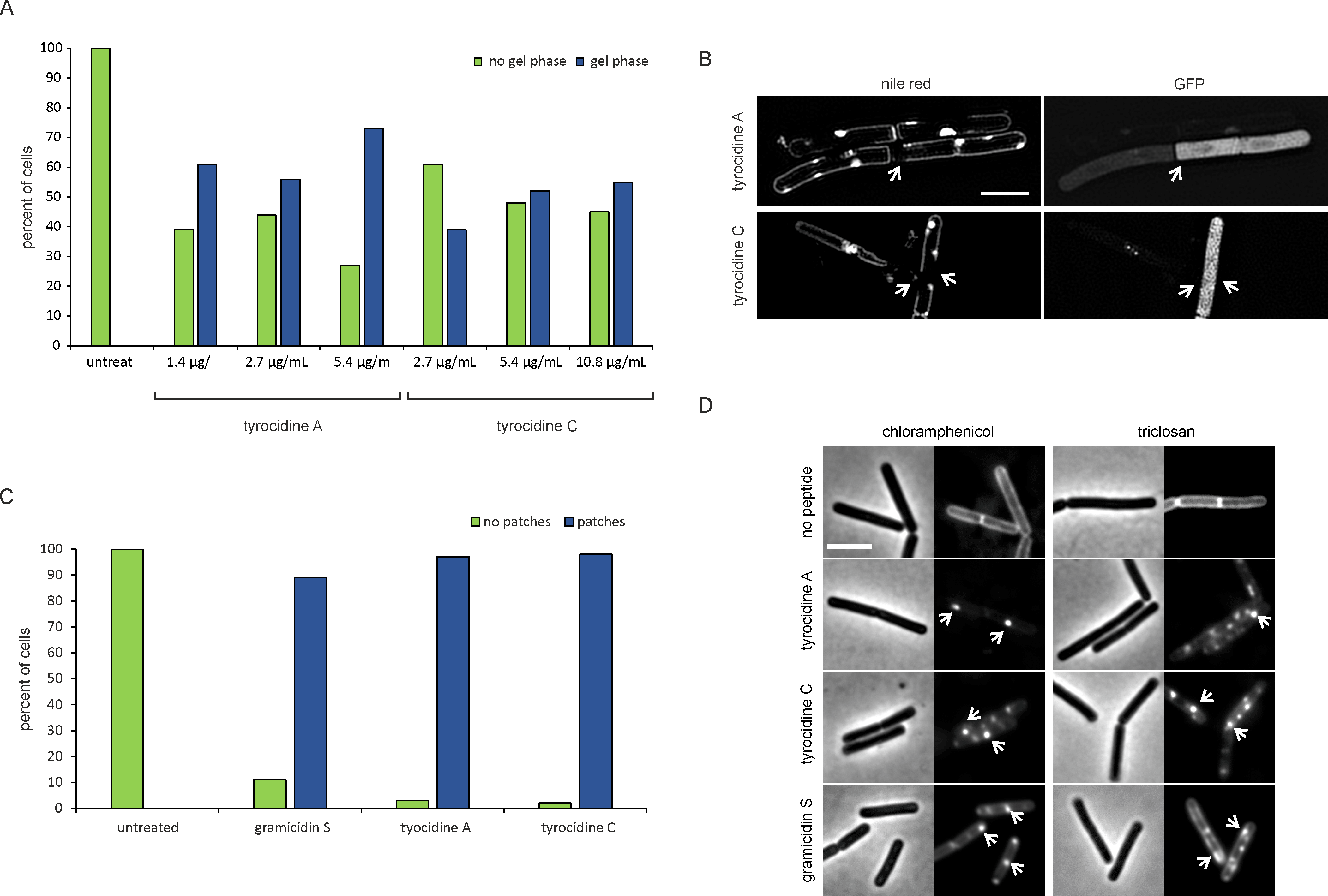

Supplement: FIG S3 [file mbo005184098sf3.tif]

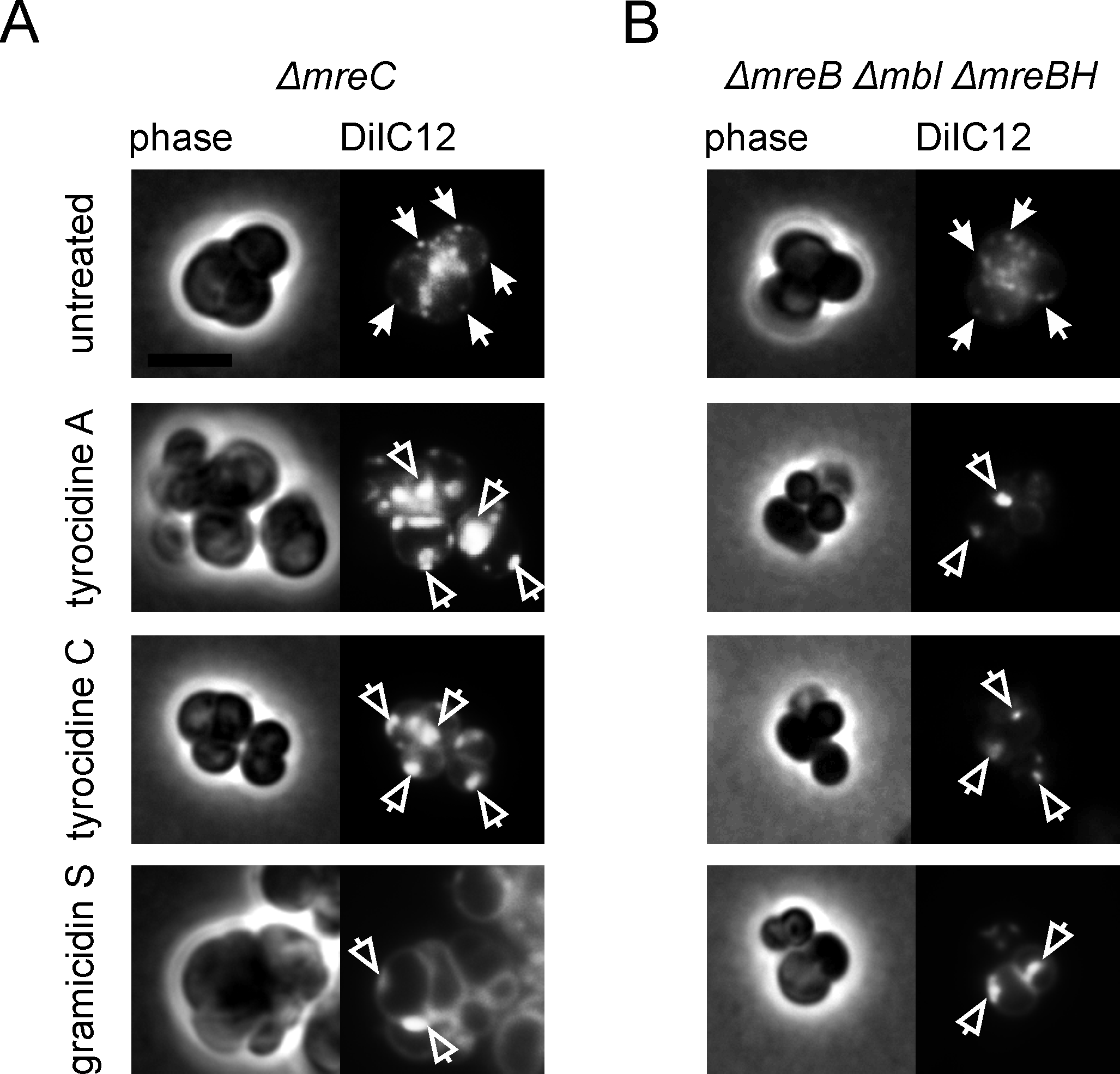

Supplement: FIG S4 [file mbo005184098sf4.tif]

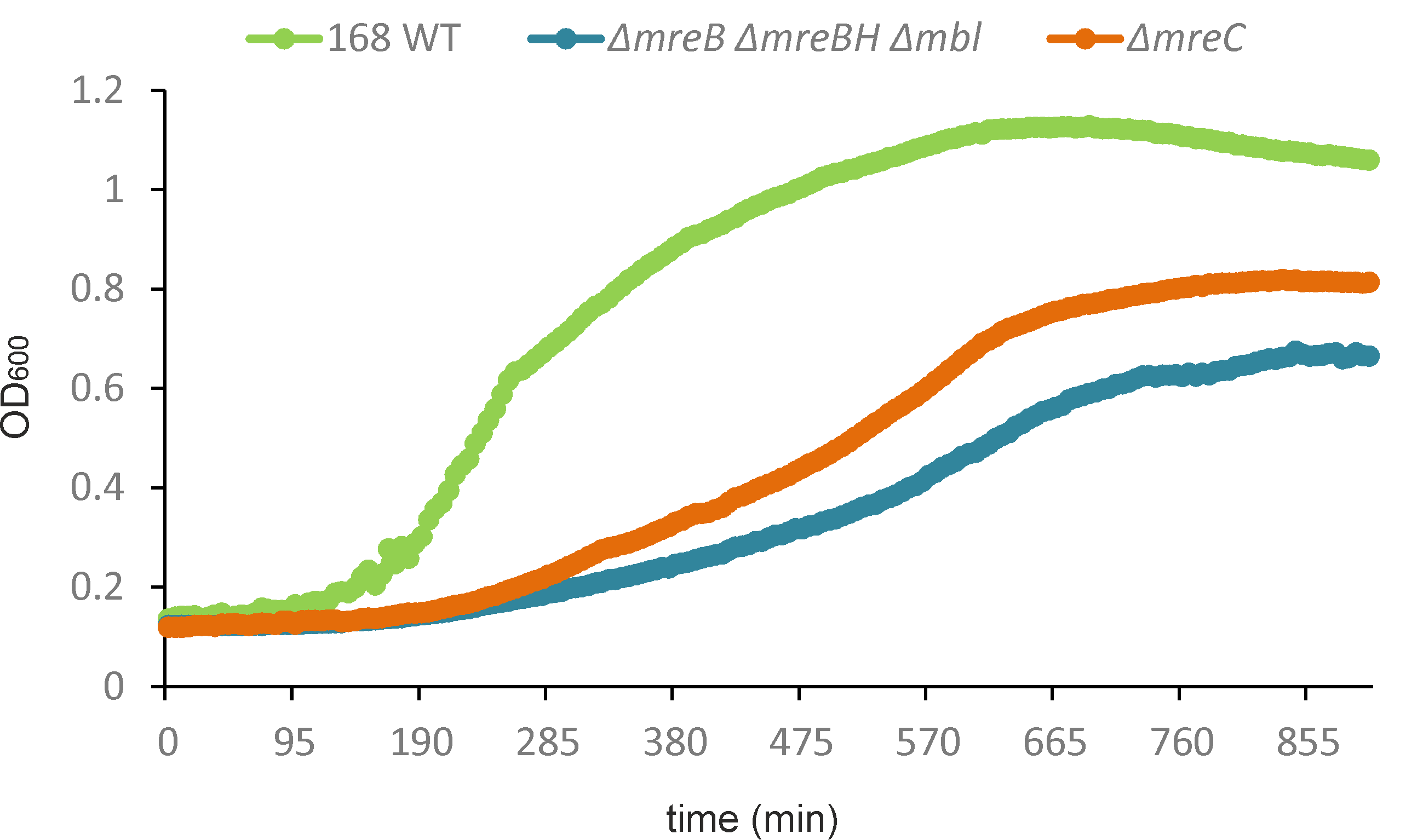

Supplement: FIG S6 [file mbo005184098sf6.tif]

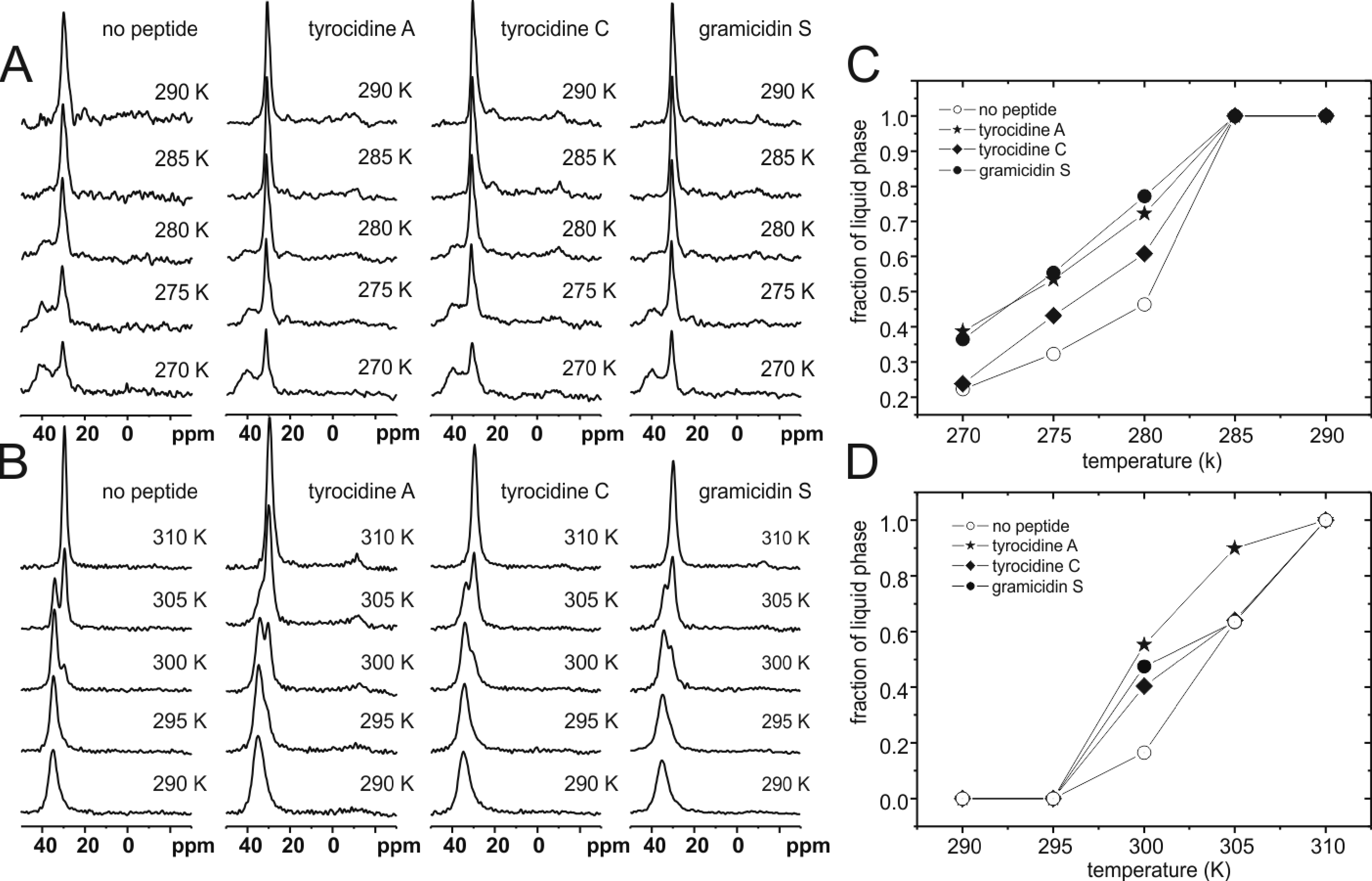

Supplement: FIG S7 [file mbo005184098sf7.tif]
