# Supplementary material for: The Multifaceted Antibacterial Mechanisms of the Pioneering Peptide Antibiotics Tyrocidine and Gramicidin S
Source: mBio. 2018 Oct 9;9(5):e00802-18. doi: 10.1128/mBio.00802-18 (PMC6178620; doi:10.1128/mBio.00802-18)
Supplement: TABLE S1 [file mbo005184098st1.docx]

**Table S1:** *B. subtilis* strains used in this study. *gfp*: green-fluorescent protein, *mgfp*: monomeric *gfp*; *sfgfp*: superfolder *gfp*, *msfgfp*: monomeric superfolder *gfp; yfp:* yellow-fluorescent protein.

| **Strain** | **genotype** | **induction/supplementation** | **reference** |
| --- | --- | --- | --- |
| 168 | *trpC2* | - | (103) |
| 1048 | *cat rpoC-gfp Pxyl-‘rpoC* | 1% xylose | (104) |
| 1049 | *amyE::spc Pxyl-rpsB-gfp* | 1% xylose | (104) |
| 3481 | *Ωneo3427* Δ*mreC* | 20 mM MgSO_4_ | (105) |
| 4277 | *Ωneo3427 ∆mreB ∆mbl::cat ∆mreBH::erm ∆rsgI::spc* | 20 mM MgSO_4_ | (106) |
| BS23 | *atpA-gfp Pxyl-′atpA cat* | 0.1% xylose | (107) |
| BS121 | *sdhA-gfp Pxyl-‘sdhA cat* | 0.1% xylose | (107) |
| bSS82 | *amyE::spc PrpsD-gfp* | - | (108) |
| DL1295 | *amyE::spc floT-yfp* | - | (53) |
| DL1367 | *amyE::spc yqfA-gfp* (*yqfA=floA*) | - | (57) |
| HM160 | *kan spo0J-gfp* | - | (47) |
| HM771 | *dnaN::gfp-dnaN cat cat* (MS104 into 168) | - | (109) |
| HS63 | *amyE::spc Pxyl-divIVA-msfgfp* | 0.5% xylose | (108) |
| LH131 | *amyE::spc Pxyl-gfp-minD* | 0.1% xylose | (59) |
| MW10 | *amyE::spc Pxyl-gfp-mreB* | 0.3% xylose | (59) |
| TNVS29D | *amyE::spc-*Pxyl-*sfgfp-plsX* | 0.5% xylose | (59) |
| TNVS175 | *amyE::spc-Pxyl-murG-msfgfp* | 0.05% xylose | (59) |
| TNVS284 | *amyE::spc-Pxyl-mraY-msfgfp* | 0.1% xylose | (59) |
| UG10 | *amyE::spc Pxyl-recA-mgfp* | 0.5% xylose | (61) |
