# Supplementary material for: The Multifaceted Antibacterial Mechanisms of the Pioneering Peptide Antibiotics Tyrocidine and Gramicidin S
Source: mBio. 2018 Oct 9;9(5):e00802-18. doi: 10.1128/mBio.00802-18 (PMC6178620; doi:10.1128/mBio.00802-18)
Supplement: TEXT S1 [file mbo005184098s1.docx]

**Text S1: MreB is not required to establish RIFs**

A curious observation that we made was that the untreated triple *mreB* mutant (Δ*mreB,* Δ*mreBH,* Δ*mbl*) still showed RIFs (Figure S5). This was surprising since in a previous study we found that the triple *mreB* mutant is devoid of RIFs and concluded that MreB is responsible for generating these fluid microdomains (1). After extensive testing it appeared that the growth phase influences the formation of RIFs (Figure S6). It appeared that RIFs become microscopically visible during exponential growth in both wild type cells and the *mreB* triple deletion mutant and disappear in the stationary phase (Figure S6), demonstrating that not the presence of MreB but the growth phase determines the occurrence of RIFs. The Δ*mreB,* Δ*mreBH,* Δ*mbl* mutant grows slower, enters exponential phase later, and reaches a lower maximum OD than the wild type strain (Figure S6), explaining why no RIFs were observed in our earlier study. The *mreC* mutant grows slightly better than the *mreB* triple mutant (Figure S7), providing an explanation why RIFs have been observed in the *mreC* deletion strain but not in the Δ*mreB,* Δ*mreBH,* Δ*mbl* mutant in our previous study (1). However, since MreB clearly co-localizes with RIFs, and the membrane potential dissipation -induced aggregation/clustering of fluid lipid does require MreB (1), we can conclude that MreB is involved in organizing RIFs along the lateral cell axis, but is not essential for making them.

**References:**

1. **Strahl H**, **Burmann F**, **Hamoen LW**. 2014. The actin homologue MreB organizes the bacterial cell membrane. Nat Commun **5**:3442.
