# Supplementary material for: The Multifaceted Antibacterial Mechanisms of the Pioneering Peptide Antibiotics Tyrocidine and Gramicidin S
Source: mBio. 2018 Oct 9;9(5):e00802-18. doi: 10.1128/mBio.00802-18 (PMC6178620; doi:10.1128/mBio.00802-18)
Supplement: TEXT S2 [file mbo005184098s2.docx]

**Text S2: Material and Methods**

Preparation of antimicrobial peptides

Tyrocidine A and C were purified according to Rautenbach *et al*. (1) from tyrothricin (Sigma-Aldrich, St Louis, USA) or from a culture extract as described by Troskie *et al*.(2). The chemical purity of tyrocidine A and C was determined as >95% with high resolution electrospray mass spectrometry linked to ultraperformace liquid chromatography as described by Troskie *et al.* (2)*.* MP196 was synthesized by solid phase synthesis as described by Chantson *et al*. (3). Daptomycin was purchased from Novartis. Benzyl alcohol, gramicidin S and CCCP were purchased from Sigma Aldrich at the highest possible purity. Tyrocidine A and C were dissolved in 30% ethanol to give 2 mg/mL stock solutions and then diluted with sterile water to 1 mg/mL working solutions. Tyrocidines were freshly dissolved immediately prior to each experiment. Benzyl alcohol was stored as argon-covered aliquots at -20 °C and diluted with DMSO to a 5 M working solution at the day of use. Gramicidin S, CCCP, and MP196 were dissolved in DMSO. Daptomycin was dissolved in sterile water. These antibiotics were stored as 10 mg/mL stock solutions at -20 °C.

Bacterial strains and growth conditions

A list of *B. subtilis* strains used in this study is displayed in Table S1. All strains were grown at 30 °C under steady agitation in Luria Bertani broth (LB). Strains 3481 and 4277 were grown in LB supplemented with 20 mM MgSO_4_. Growth experiments were performed in 96-well plates using a BioTek Synergy MX plate reader. Cells were inoculated to an OD_600_ of 0.05 and grown until an OD_600_ of 0.3 before splitting the culture and treating aliquots with different compound concentrations. Unless otherwise stated the following concentrations were used: 5.4 µg/mL tyrocidine A, 2.7 µg/mL tyrocidine C, 1 µg/mL gramicidin S, 100 µM CCCP, 10 µg/mL MP196, 1 µg/mL daptomycin, and 50 mM benzyl alcohol. For daptomycin treatment, cells were grown in LB supplemented with 1.25 mM CaCl_2_.

Determination of minimal inhibitory concentrations and growth experiments

Minimal inhibitory concentrations (MICs) against *B. subtilis* 168 and *S. aureus* 8325 were determined in LB in a standard serial dilution assay in 96-well format. LB, containing serial dilutions of compounds, was inoculated with 5x10^5^ CFU/mL and incubated at 37 °C for 16 h under constant shaking in a BioTek Synergy MX plate reader. The MIC was defined as the lowest compound concentration inhibiting bacterial growth. In order to examine the mode of action of the tyrocidines and gramicidin S in *B. subtilis in vivo*, we additionally determined the MIC of the peptides in a test tube assay to mimic the exact same conditions for further mode of action experiments (Figure 9A). At this concentration, the compounds caused an immediate growth stop of exponential phase *B. subtilis* cultures, whereby tyrocidine A and C induced cell lysis after approximately 20 min (Figure 1D). In order to analyze the immediate growth-inhibiting effects of the peptides and not pleiotropic lysis effects, we performed further experiments after 10 min treatment time.

Fluorescence light microscopy

Fluorescence light microscopy was performed using a Nikon Eclipse Ti equipped with a CFI Plan Apochromat DM 100x oil objective, an Intensilight HG 130 W lamp, a C11440-22CU Hamamatsu ORCA camera, and NIS elements software, version 4.20.01. Images were analyzed using ImageJ (National Institutes of Health) v.1.48. All strains were grown in LB at 30 °C under steady agitation in the presence of appropriate inducer concentrations (Table S1). Antibiotics were added at an OD_600_ of 0.3 and images were taken after 10 min of antibiotic treatment. Cells were immobilized on 1.2% agarose-covered slides (4). Cell wall integrity was probed with an acetic acid/methanol fixation method as described previously (5). Membranes were stained with 2 µg/mL FM5-95 for 10 min, 1 µg/mL mitotracker green for 2 min, or 1 µg/mL Nile red for 2 min. Nucleoids were stained with 1 µg/mL DAPI for 2 min. DiIC12 staining and laurdan microscopy were performed as described previously(6). Analysis of laurdan microscopy images was performed with Image J using the ‘calculate GP’ plugin (7).

Transmission electron microscopy

*B. subtilis* 168 was grown in LB until early exponential phase and subsequently treated with the peptides for 10 min. Cells were then placed on an agarose patch, allowed to dry for 2 min, and subsequently fixed with 5% glutaraldehyde in 0.1 M cacodylate buffer (pH 7.4) for 20 min. Agarose-embedded samples were washed three times with 0.1 M cacodylate buffer (pH 7.4) and then stained with a 1:1 mixture of osmium tetroxide (1%) and K_3_[Ru(III)(CN)_6_] (1%) for 30 min, followed by three times washing with water. Samples were then dehydrated in an incubation series with rising concentrations of ethanol as follows: 5 min 30% ethanol, 5 min 50% ethanol, 15 min 70% ethanol, 60 min 80% ethanol, 15 min 90% ethanol, 15 min 96% ethanol, 15 min 100% ethanol, 30 min 100% ethanol (water-free), 5 min propylene oxide, 30 min 1:1 EPON/propylene oxide, 30 min 2:1 EPON/propylene oxide. Samples were then covered with fresh EPON, incubated over night at room temperature, and subsequently allowed to polymerize at 65 °C for 36 h prior to ultrathin sectioning. Pictures were taken with a JEOL 1010 transmission electron microscope at an electron voltage of 80 kV.

Propidium iodide assay

Permeability for the large fluorescent molecule propidium iodide was quantified using a BioTek Synergy MX platereader. Cells were grown until an OD_600_ of 0.3 and antibiotics were added simultaneously with 13.3 µg/mL propidium iodide (1 mg/mL stock in DMSO). After 5 min, cells were washed twice with LB (short spin) and fluorescence was measured using 535 nm excitation and 617 nm emission wavelengths.

Electrophysiological analysis of the pore forming ability using planar lipid bilayer arrays

The phospholipids POPG and POPE (Avanti polar lipids) were dissolved in octane and combined in a 3:1 POPG/POPE molar ratio providing a total lipid concentration of 10 mg/mL. Planar lipid bilayer membranes were formed by remote liquid spreading in an Orbit 16 automated parallel bilayer platform (Nanion Technologies) on the microelectrode cavity array (MECA) chip as described earlier (8). Bilayer formation was controlled with the internal multichannel patch-clamp amplifier (Triton-16, Tecella) of Orbit-16 running under TecellaLab software. All experiments were conducted at 25 °C in buffer containing 150 mM KCl, 10 mM Tris, 1 mM EDTA, pH 7.4. After lipid bilayer formation, the holding potential was set to +100 mV (*trans*-side). The *cis*-side was set to electrical ground. The stability of the formed membranes was checked for at least 3 min. No increase in current was recorded in the controls. Peptide stock solutions at 100 µg/mL in 50% ethanol were pre-diluted to a final ethanol concentration below 0.5% with measurement buffer, before being added to the *cis* side of the lipid bilayers. Final peptide concentrations sufficient to induce fluctuations of the membrane current were in the range 1-20 nM for tyrocidine A and tyrocidine C and 1-5 µM for gramicidin S. Analysis of the single channel current traces was performed by computing histograms of current transitions from the original current-time traces with ClampFit 10.5 software from Molecular Devices. All current traces shown in the figures were further filtered using a digital Bessel low-pass filter with a cutoff frequency of 250 Hz.

Membrane potential measurements

The membrane potential was measured with the fluorescent probe DiSC(3)5 as described earlier (6). Measurements were carried out in black polystyrene microtiter plates (Labsystems) in a BioTek Synergy MX plate reader, using 610 nm excitation and 660 nm emission wavelengths.

Solid state nuclear magnetic resonance spectrometry

Lipid stock solutions were prepared in chloroform as follows: POPE at 100 mg/mL and POPG at 50 mg/mL. Lipids were subsequently combined in a specific molar ratio of 3:1 POPG/POPE to a combined weight of 10 mg to mimic the Gram-positive membrane composition. Macroscopically oriented lipid bilayers in the presence and absence of the antimicrobial peptides (2% mol/mol) were prepared by adding a methanol solution of 1 mg/mL peptide to the lipid solution to obtain a 1:50 peptide-to-lipid molar ratio (9). Negative control lipid samples only received methanol. The resulting suspension was brought to 400 µL with chloroform to ensure all peptides and lipids were dissolved. Nitrogen gas was then utilized to decrease the volume, after which each solution was placed and dried in approximate equal proportions on three ultrathin cover glasses (18 mm × 9 mm, Marienfeld). The slides were then placed in high vacuum overnight to ensure that all solvent was removed. Subsequently, the slides were transferred into a sealed chamber kept at 93% humidity by a saturated solution of KNO_3_. The following day, the slides where stacked and equilibrated for another night at 93% relative humidity. Stacked slides where wrapped in Teflon tape before being sealed in plastic. This procedure ensures that lipids are well-hydrated without excess water being present in the sample, improving resolution and quantitative information from the recorded spectra. Furthermore, the absence of bulk water forces all peptides to associate with the membrane, allowing working with a more exact peptide-to-lipid ratio without detailed knowledge of the association constants of the peptides to the particular membrane under investigation. For spectral acquisition the sample was placed into the flattened coil of a static triple resonance probe (10) with the glass plate normal parallel to the magnetic field of a 300 MHz Advance NMR spectrometer (Bruker). Proton-decoupled ^31^P solid-state NMR spectra were recorded at 270 K, 290 K and 310 K using a Hahn echo pulse sequence (11) using the following parameters: ^31^P B_1_ field 42 kHz and an echo time of 20 µs. The dwell time was 13.7 µs for 512 data points resulting in a spectral width of 36.5 kHz. The number of scans was typically 4096 with a recycle delay of 5 s. The reference scale was based on 85% phosphoric acid set to 0 ppm.

Structured illumination microscopy

3D structured illumination microscopy (SIM) was performed using a Nikon Eclipse Ti N-SIM E microscope setup equipped with a CFI SR Apochromat TIRF 100x oil objective (NA1.49), an LU-N3-SIM laser unit, an Orca-Flash 4.0 sCMOS camera (Hamamatsu Photonics K.K.), and NIS elements Ar software. Cultures were grown, stained, and mounted on microscopy slides as described above. For SIM, mitotracker green was used as green, and Nile red as red membrane dye. Poly-dopamine-coated coverslips were used to reduce background fluorescence (4).

Time lapse microscopy

Time lapse microscopy was performed with a Nikon Eclipse Ti N-SIM E as specified above using wide field image acquisition mode. Cells were grown until early log phase in LB, mounted on agarose pads, placed into a flow chamber (ibidi), and allowed to grow out at a constant temperature (30 °C) and constant flow (0.2 mL/min) of fresh pre-warmed LB for 30 min. Then the respective compounds were added to the LB flow. Due to the high affinity of the peptides to plastic surfaces, 10-fold compound concentrations had to be used (54 µg/mL tyrocidine A, 27 µg/mL tyrocidine C). Additionally, LB was supplemented with 2 mg/mL L-dopamine to block binding of tyrocidines to the microfluidics tubing. A control picture was taken prior to addition of antibiotic. After compound addition, pictures were taken every 30 sec. For staining with FM5-95, cultures were stained with 2 µg/mL of the dye for 10 min prior to mounting the sample on the slide. FM5-95 was used for time lapse microscopy, because it does not affect the growth rate of *B. subtilis*. In order to reduce background fluorescence of FM5-95, cover slips were coated with poly-dopamine (4).

Laurdan spectroscopy

Membrane fluidity in batch culture was determined with laurdan as described before (6). Cells were grown until early exponential phase, stained with 10 µM laurdan, washed four times with PBS supplemented with 2% glucose, and resuspended in the same buffer to give an OD_600_ of 0.3. Readings were taken every 2 min. Antibiotics were added after 4 min and fluorescence was measured over an additional 30 min. Laurdan fluorescence was measured in a BioTek Synergy MX plate reader using 350 nm excitation and 460 and 500 nm emission wavelengths. Laurdan generalized polarization (GP) values were calculated with the formula (I_460_-I_500_)/(I_460_+I_500_).

Activity assay against non-growing cells

*B. subtilis* 168 and *S. aureus* 8325 were aerobically grown over night at 37 °C. These stationary phase cultures were then incubated with 10-40x MIC of the respective compounds for 9 h at 37 °C under continuous shaking followed by CFU determination by plating dilution series on LB plates without antibiotics. Antibiotic concentrations were adjusted to the higher number of cells present in stationary phase overnight cultures, compared to MIC inoculation based on optical density measurements.

**References**

1. **Rautenbach M**, **Vlok NM**, **Stander M**, **Hoppe HC**. 2007. Inhibition of malaria parasite blood stages by tyrocidines, membrane-active cyclic peptide antibiotics from Bacillus brevis. Biochim Biophys Acta **1768**:1488–1497.

2. **Troskie AM**, **de Beer A**, **Vosloo JA**, **Jacobs K**, **Rautenbach M**. 2014. Inhibition of agronomically relevant fungal phytopathogens by tyrocidines, cyclic antimicrobial peptides isolated from Bacillus aneurinolyticus. Microbiology **160**:2089–2101.

3. **Chantson JT**, **Falzacappa MVV**, **Crovella S**, **Metzler-Nolte N**. 2006. Solid-phase synthesis, characterization, and antibacterial activities of metallocene-peptide bioconjugates. ChemMedChem **1**:1268–1274.

4. **te Winkel JD**, **Gray DA**, **Seistrup KH**, **Hamoen LW**, **Strahl H**. 2016. Analysis of antimicrobial-triggered membrane depolarisation using voltage sensitive dyes. Front Cell Dev Biol **4**:29.

5. **Wenzel M**, **Kohl B**, **Münch D**, **Raatschen N**, **Albada HB**, **Hamoen L**, **Metzler-Nolte N**, **Sahl HG**, **Bandow JE**. 2012. Proteomic response of Bacillus subtilis to lantibiotics reflects differences in interaction with the cytoplasmic membrane. Antimicrob Agents Chemother **56**:5749–5757.

6. **Müller A**, **Wenzel M**, **Strahl H**, **Grein F**, **Saaki TN V**, **Kohl B**, **Siersma T**, **Bandow JE**, **Sahl H-G**, **Schneider T**, **Hamoen LW**. 2016. Daptomycin inhibits cell envelope synthesis by interfering with fluid membrane microdomains. Proc Natl Acad Sci USA **113**:E7077–E7086.

7. **Saeloh D**, **Tipmanee V**, **Jim KK**, **Dekker MP**, **Bitter W**, **Voravuthikunchai SP**, **Wenzel M**, **Hamoen LW**. 2018. The novel antibiotic rhodomyrtone traps membrane proteins in vesicles with increased fluidity. PLoS Pathog **14**:e1006876.

8. **del Rio Martinez JM**, **Zaitseva E**, **Petersen S**, **Baaken G**, **Behrends JC**. 2015. Automated formation of lipid membrane microarrays for ionic single-molecule sensing with protein nanopores. Small **11**:119–125.

9. **Aisenbrey C**, **Bertani P**, **Bechinger B**. 2010. Solid-state NMR investigations of membrane-associated antimicrobial peptides. Methods Mol Biol **618**:209–233.

10. **Bechinger B**, **Opella SJ**. 1991. Flat-coil probe for NMR spectroscopy of oriented membrane samples. J Magn Reson **95**:585–588.

11. **Rance M**, **Byrd RA**. 1983. Obtaining high-fidelity spin-12 powder spectra in anisotropic media: Phase-cycled Hahn echo spectroscopy. J Magn Reson **52**:221–240.
